# The myeloid lineage is required for the emergence of a regeneration-permissive environment following *Xenopus* tail amputation

**DOI:** 10.1242/dev.185496

**Published:** 2020-02-05

**Authors:** Can Aztekin, Tom W. Hiscock, Richard Butler, Francisco De Jesús Andino, Jacques Robert, John B. Gurdon, Jerome Jullien

**Affiliations:** 1 Wellcome Trust/Cancer Research UK Gurdon Institute, University of Cambridge, Cambridge, CB1 2QN, UK; 2 Department of Zoology, University of Cambridge, Cambridge, CB2 3EJ, UK; 3 Cancer Research UK Cambridge Institute, University of Cambridge, Cambridge, CB2 0RE, UK; 4 Department of Microbiology & Immunology, University of Rochester Medical Center, Rochester, NY 14642, USA; 5 Nantes Université, Inserm, Centre de Recherche en Transplantation et Immunologie, UMR 1064, ITUN, F-44000 Nantes, France

**Keywords:** *Xenopus*, Tail regeneration, Myeloid lineage, Inflammation, Macrophages, Neutrophils

## Abstract

Regeneration-competent vertebrates are considered to suppress inflammation faster than non-regenerating ones. Hence, understanding the cellular mechanisms affected by immune cells and inflammation can help develop strategies to promote tissue repair and regeneration. Here, we took advantage of naturally occurring tail regeneration-competent and -incompetent developmental stages of *Xenopus* tadpoles. We first establish the essential role of the myeloid lineage for tail regeneration in the regeneration-competent tadpoles. We then reveal that upon tail amputation there is a myeloid lineage-dependent change in amputation-induced apoptosis levels, which in turn promotes tissue remodelling, and ultimately leads to the relocalization of the regeneration-organizing cells responsible for progenitor proliferation. These cellular mechanisms failed to be executed in regeneration-incompetent tadpoles. We demonstrate that regeneration incompetency is characterized by inflammatory myeloid cells whereas regeneration competency is associated with reparative myeloid cells. Moreover, treatment of regeneration-incompetent tadpoles with immune-suppressing drugs restores myeloid lineage-controlled cellular mechanisms. Collectively, our work reveals the effects of differential activation of the myeloid lineage on the creation of a regeneration-permissive environment and could be further exploited to devise strategies for regenerative medicine purposes.

## Abstract (precis)

**Summary:** *Xenopus* tail regeneration requires a hierarchy of cellular events initiated by the myeloid lineage and culminating in the mobilization of regeneration-organizing cells.

## INTRODUCTION

Regeneration-competent vertebrates are considered to have a limited immune system development and competency (Julier et al., 2017). However, the immune system has also been demonstrated to influence regeneration positively in various scenarios, e.g. in appendages (Godwin et al., 2013; Petrie et al., 2014) and in cardiac tissue (Godwin et al., 2017; Lavine et al., 2014), and in several species, e.g. salamander (Godwin et al., 2017, 2013), zebrafish (Morales and Allende, 2019; Nguyen-Chi et al., 2017; Petrie et al., 2014) and mouse (Lavine et al., 2014; Simkin et al., 2017). In particular, myeloid lineage-driven activation and rapid resolution of inflammation have been suggested to influence the outcome of regeneration (Godwin, 2014; Mescher et al., 2017). Regeneration-competent animals are proposed to efficiently suppress damage-induced inflammation. By contrast, regeneration-incompetent animals have a prolonged inflammatory phase that results in an impairment of extracellular matrix remodelling mediated by excessive collagen deposition and scar formation (Godwin, 2014). In such animals, suppression of inflammation improves injury repair and regeneration (Chu et al., 2019; Gensel et al., 2017). The positive effect of the myeloid lineage on tissue repair and regeneration, mostly characterized in mammalian models, was shown to be mediated through promotion of repair processes via extracellular matrix (ECM) remodelling, histolysis, vascularization, and apoptotic cell clearance (Koh and DiPietro, 2011). However, an overall understanding of the cellular events controlled by the inflammatory states and their effect in animals with high regenerative capabilities is lacking.

*Xenopus laevis* tadpoles can regenerate their tails throughout development but temporarily lose this ability at certain developmental stages (Slack et al., 2004). Previously, we showed that this loss is caused by failure to mobilize a signal-centre cell population, the regeneration-organizing-cells (ROCs) (Aztekin et al., 2019). In regeneration-competent tadpoles, ROCs relocate from the body to the amputation plane to form a specialized wound epidermis, and by secreting a cocktail of growth factors they can increase progenitor cell proliferation. However, the contribution of the myeloid lineage to this process in regeneration-competent stages is not known. Moreover, unlike in other regeneration models in which the presence of the immune system is required for regeneration, decreasing the immune cells was suggested to boost regeneration in naturally occurring regeneration-incompetent tadpoles (Fukazawa et al., 2009). Nonetheless, how such perturbations can reinstate regeneration competency remains unclear.

Here, we first demonstrated the essential role of the myeloid lineage in regeneration-competent tadpoles using complementary myeloid lineage-depletion methods. We then functionally tested which cellular mechanisms are controlled by the myeloid lineage, the relationship between identified cellular mechanisms, and their requirement for successful regeneration. Lastly, we characterized the behaviour of the myeloid lineage in naturally occurring regeneration-competent and -incompetent tadpoles. Altogether, our investigation reveals the hierarchy of cellular mechanisms controlled by the myeloid lineage that is responsible for the emergence of a regeneration-permissive environment. These findings could be exploited to boost injury repair and regeneration in regeneration-incompetent animals.

## RESULTS

### The myeloid lineage is required for *Xenopus* tail regeneration

**Fig. 1.**
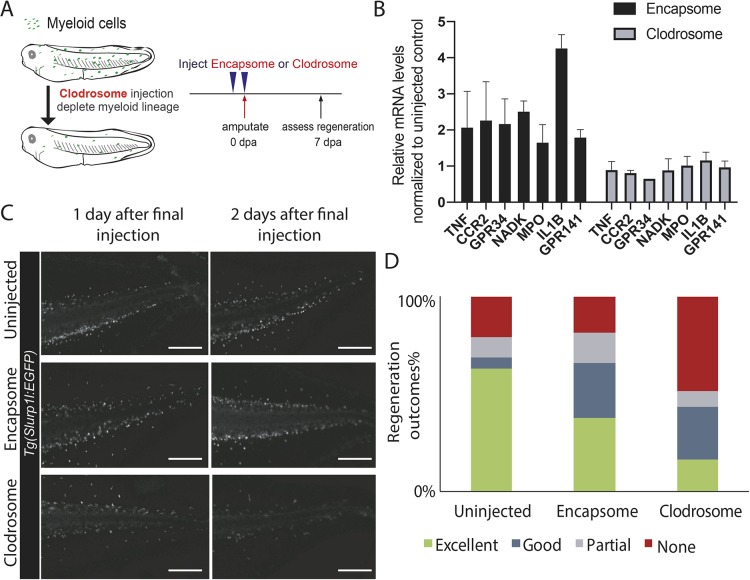
**Depleting the myeloid lineage impedes *Xenopus* tail regeneration.** (A) Experimental design for assessing the effect of myeloid lineage depletion on regeneration. Regeneration-competent tadpoles were injected with either control (Encapsome), or clodronate-containing lipids (Clodrosome) before tail amputation. Regeneration efficiency was assessed at 7 days post-tail amputation (dpa). (B) Clodrosome injection decreases myeloid gene expression compared with Encapsome injection. Expression of myeloid lineage genes after Encapsome or Clodrosome injections was assessed by RT-qPCR analysis on amputated tails. All values were normalized to that of the uninjected controls. *n*≥3 biological replicates for each gene expression quantification, error bars represent s.e.m. (C) Representative images of *Tg(slurp1l:EGFP)* tadpoles post Encapsome or Clodrosome injection. A decrease in the *slurp1l:EGFP* myeloid lineage signal is particularly seen at dorsal and ventral vein regions. Scale bars: 250 µm. (D) Regeneration outcomes at 7 dpa in uninjected, Encapsome-injected or Clodrosome-injected regeneration-competent tadpoles. Samples were obtained from three biological replicates: uninjected *n*=30; Encapsome *n*=34; Clodrosome *n*=46.

We first asked whether the myeloid lineage is required for regeneration by injecting clodronate-containing liposomes into the ventral vein area of tadpoles (Fig. 1A) then assessing regeneration ability after tail amputation. When engulfed by phagocytes, clodronate induces cell death and leads to myeloid cell depletion (van Rooijen and Hendrikx, 2010). Upon clodronate-containing liposome injection, we observed a reduced number of myeloid lineage cells and reduced expression of genes associated with myeloid lineage cells at the time of tail amputation (Fig. 1B,C, Fig. S1A). Moreover, upon tail amputations, these tadpoles had a reduced tail regeneration compared with control, Encapsome-injected animals (Fig. 1D, Fig. S1B), suggesting the myeloid lineage is required for regeneration. To independently assess the role of myeloid lineage in regeneration, we generated F_0_ transgenic tadpoles with a drug-inducible myeloid cell ablation construct in which the *Escherichia coli Nitroreductase* (*NTR*) gene is under the control of the myeloid marker *slurp1l* promoter. When metronidazole (MTZ) is added, NTR-expressing cells are killed (Martinez-De Luna and Zuber, 2018) (Fig. S2A) as detected at the time of tail amputation. Upon removal of *slurp1l*-expressing cells, we again observed reduced regeneration (Fig. S2B-D). However, upon tail amputation, we also observed *slurp1l* promoter activation in non-myeloid lineages, including ROCs (Fig. S2E). Hence, in later stages of regeneration, this method might ablate important non-myeloid cell types. To test further the requirement of the myeloid lineage for regeneration, we generated F_0_ tadpoles that have defective myeloid lineage development but show no lethality induced by mosaic *spib* gene knockout (Costa et al., 2008). Perturbing the *spib* gene reduced the myeloid gene expressions at the time of tail amputation (Fig. S3A-C). When these tadpoles were assessed for tail regeneration ability, we observed reduced growth of regenerated tails, indicative of delay in the regenerative programme (Fig. S3D,E). Although the myeloid lineage of F_0_ tadpoles was decreased at the time of tail amputation, we observed restoration of myeloid gene expressions at the end of regeneration (Fig. S3F), presumably because of the mosaic nature of the knockout. Taken together, these complementary approaches indicate that the myeloid lineage is required for regeneration.

### The myeloid lineage is required for tissue remodelling, reduced apoptosis, and ROC mobilization in regeneration-competent tadpoles

Successful regeneration is associated with tissue/ECM remodelling (Contreras et al., 2009), regulated apoptosis levels (Tseng et al., 2007), and ROC mobilization (Aztekin et al., 2019). Therefore, focusing on regeneration-competent tadpoles, we next asked if these events require the myeloid lineage.

To assess quantitatively the level of tissue remodelling, we calculated a ‘remodelling index’ as the ratio of the length of the whole amputation plane to that of the posterior somitic tissue (Fig. S4A-C). We based this assessment on previously reported changes occurring at the amputation plane. Particularly, as previously reported (Beck et al., 2003), at 1 day post-amputation (dpa), evidence of tissue remodelling can be seen as muscle tissue degenerates and somitic tissue is reshaped at the amputation plane. Moreover, ventral and dorsal fin tissues start covering the amputation plane.

**Fig. 2.**
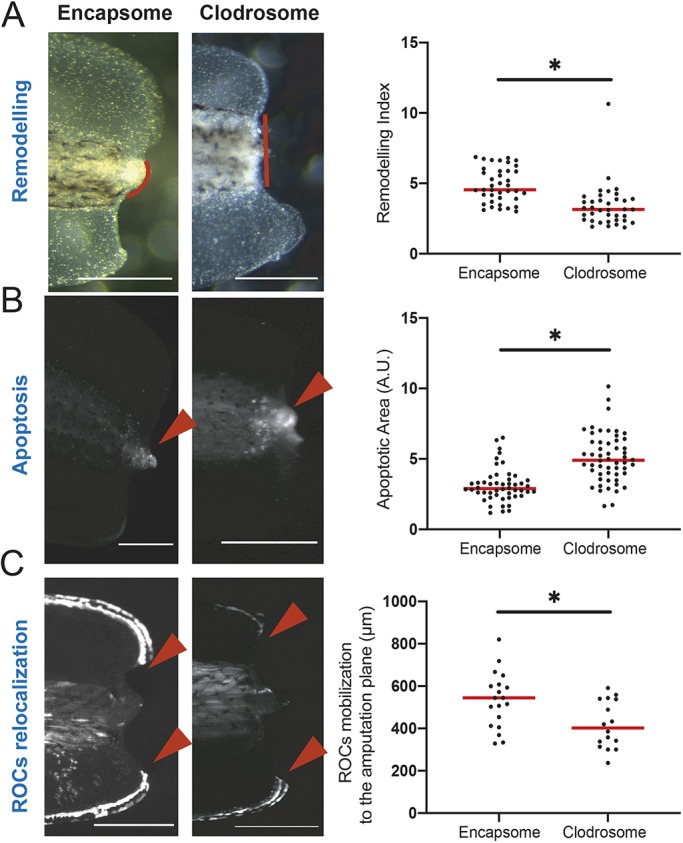
**The myeloid lineage is required for tissue remodelling, reduction of apoptosis levels, and relocalization of regeneration-organizing cells.** (A) Left: Representative images of remodelling events occurring at 1 dpa in Encapsome- or Clodrosome-injected regeneration-competent tadpoles. Red lines indicate the posterior somitic region. Post-amputation histolysis and remodelling of tissue is impaired in Clodrosome-injected tadpoles. Scale bars: 250 µm. Right: Remodelling index quantification. All samples were obtained from at least three biological replicates: Encapsome *n*=39; Clodrosome *n*=37. Student's *t*-test was used to assess statistical significance; *P<0.001. (B) Left: Representative images of apoptosis occurring at 1 dpa in Encapsome- or Clodrosome-injected regeneration-competent tadpoles. The red arrowheads indicate the location of the LysoSensor signal used to report apoptosis. Scale bars: 250 µm. Right: Apoptotic area quantification. All samples were obtained from at least three biological replicates: Encapsome *n*=49; Clodrosome *n*=53. Student's *t*-test was used to assess statistical significance; *P<0.001. A.U., arbitrary unit. (C) Left: Representative images of ROC relocalization at 1 dpa for Encapsome- or Clodrosome-injected regeneration-competent tadpoles. The 7pbin:EGFP transgenic line was used to assess ROCs relocalization. Red arrowheads show the location of the leading ROC cells. Scale bars: 250 µm. Right: ROC relocalization quantification. All samples were obtained from at least three biological replicates: Encapsome *n*=19; Clodrosome *n*=16. Student's *t*-test was used to assess statistical significance; *P<0.001. In graphs, each dot represents individual tadpoles; red line denotes median.

When the myeloid lineage was removed in regeneration-competent tadpoles, the remodelling was reduced (Fig. 2A, Fig. S6A). Furthermore, this phenotype mimicked remodelling levels that were seen in samples immediately after amputation, and at 1 dpa in regeneration-incompetent tadpoles (Fig. S4D,E). Hence, the myeloid lineage is required upstream of morphological changes associated with regeneration competency.

**Fig. 3.**
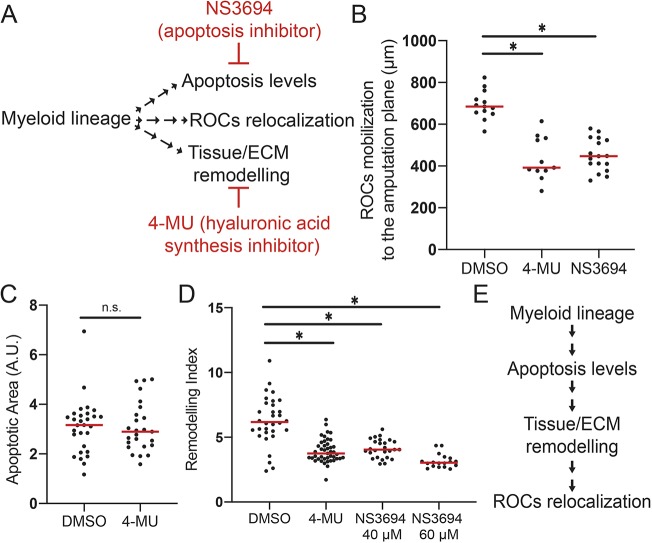
**Sequential involvement of apoptosis, tissue remodelling and regeneration-organizing cell mobilization following tail amputation.** (A) Experimental setup for assessing the relationship between the cellular mechanisms controlled by amputation-induced myeloid lineage activity. Cellular events downstream of myeloid lineage activity were assessed after drug treatments. NS3694 (apoptosis inhibitor) and 4-MU (hyaluronic acid synthesis inhibitor) were used to block apoptosis, and tissue remodelling, respectively. (B) ROC relocalization levels at 1 dpa in DMSO control, or after 4-MU or NS3694 treatment in regeneration-competent tadpoles. All samples were obtained from two biological replicates: DMSO *n*=13; 4-MU *n*=11; NS3694 *n*=17. One-way ANOVA was used to assess statistical significance; *P<0.001. (C) Apoptotic area at 1 dpa in DMSO- or 4-MU-treated regeneration-competent tadpoles. All samples were obtained from four biological replicates: DMSO *n*=29; 4-MU *n*=25. Student's *t*-test was used to assess statistical significance; *P<0.001. n.s., not significant. A.U., arbitrary unit. (D) Quantification of remodelling index at 1 dpa in DMSO-, 4-MU- or NS3694-treated regeneration-competent tadpoles. All samples were obtained from at least three biological replicates: DMSO *n*=32; 4-MU *n*: 43, NS3694 40 µM *n*=26; NS3694 60 µM *n*=18. One-way ANOVA was used to assess statistical significance; *P<0.001. In B-D, each dot represents individual tadpoles; red line denotes median. (E) Epistasis experiments suggest that the myeloid lineage controls apoptosis levels, which influence tissue/ECM remodelling through HA deposition and, finally, ROCs relocalization.

Underlying these morphological changes are alterations in the biosynthetic pathway of ECM components (Bonnans et al., 2014; Lu et al., 2011), such as that for hyaluronic acid (HA), which was previously shown to be required for regeneration (Contreras et al., 2009). Hence, we investigated whether blocking HA synthesis can phenocopy myeloid lineage-depleted tadpoles. Indeed, when HA synthesis was blocked, remodelling was decreased (Fig. 3D). We conclude that the regulation of tissue remodelling by the myeloid lineage involves control of HA deposition.

We next asked whether the level of apoptosis associated with successful regeneration is regulated by the myeloid lineage. A controlled level of apoptosis is proposed to be necessary for regeneration (Tseng et al., 2007). By using cytoplasmic acidity as an indicator of apoptosis (Fig. S5A-C), and consistent with published results (Tseng et al., 2007), we observed that at 1 dpa regeneration-incompetent tadpoles have more apoptotic cells compared with regeneration-competent tadpoles (Fig. S5D). Removal of the myeloid lineage from regeneration-competent tadpoles led to increased apoptosis (Fig. 2B, Fig. S6B), indicating that the myeloid cells are involved in the regulation of apoptotic cell level, possibly through apoptotic cell clearance (Gordon and Plüddemann, 2018; Zhong et al., 2018). Hence, amputation-induced apoptosis, and its clearance via myeloid lineage activity, is likely to be crucial for regeneration.

As the myeloid lineage controls tissue/ECM remodelling and apoptosis levels, we asked if these events create a regeneration-permissive environment contributing to ROC relocalization to the amputation plane. Indeed, removal of myeloid lineage (Fig. 2C, Fig. S6C), blocking the HA pathway (Fig. 3A,B) or blocking apoptosis (Fig. 3A,B) interfered with ROC mobilization, indicating that all these events are upstream of ROC relocalization. Next, to understand the relationship between remodelling and apoptosis, we blocked HA synthesis and assessed apoptosis levels. Blocking HA synthesis did not significantly alter apoptosis levels (Fig. 3C). Conversely, we observed that blocking apoptosis reduced tissue remodelling (Fig. 3D). Together, these results indicate that the myeloid lineage controls apoptosis levels, which is an upstream event of HA deposition-mediated tissue remodelling, itself leading to ROC relocalization (Fig. 3E).

### The ratio of reparative to inflammatory myeloid cells following amputation is associated with regeneration competency

**Fig. 4.**
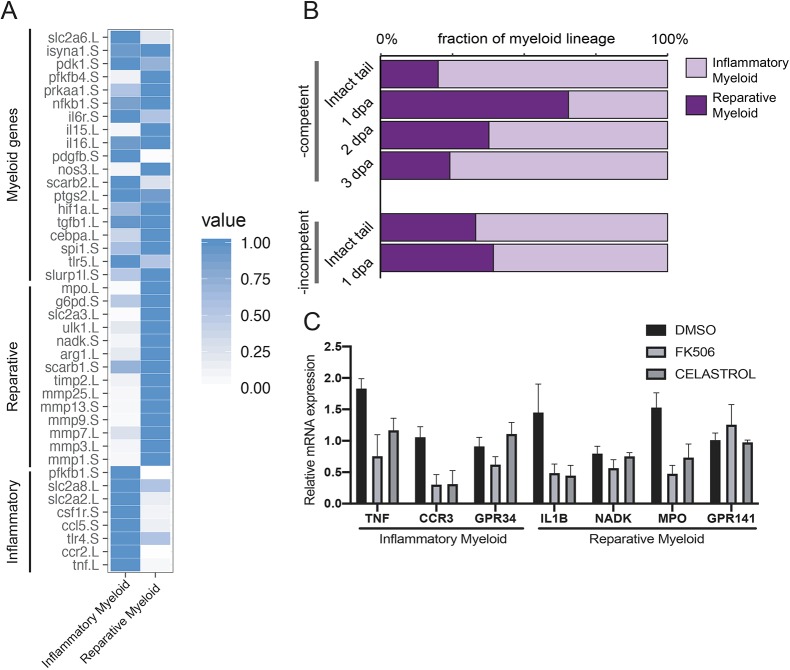
**A post-amputation shift from inflammatory to reparative myeloid activity characterizes regeneration-competent tadpoles.** (A) Heat map showing known myeloid, reparative and inflammatory gene expression levels in the *Xenopus* tail cell atlas myeloid cell clusters. Values for each gene were normalized to that of the highest expressing cluster. Data were re-analysed from Aztekin et al. (2019). (B) Relative abundance of inflammatory and reparative myeloid lineage cells post-amputation in regeneration-competent and -incompetent tadpoles. Data were re-analysed from Aztekin et al. (2019). (C) Myeloid cell cluster 1 and 2 gene expressions were tested 1 day after treatment of regeneration-incompetent tadpoles with the immune-suppressing drugs FK506 or Celastrol. *n>*3 biological replicates for each gene expression quantification; error bars represent s.e.m.

In contrast to our results with regeneration-competent tadpoles in which the myeloid lineage is required for regeneration, decreasing immune cell number in regeneration-incompetent tadpoles was previously suggested to rescue regeneration (Fukazawa et al., 2009). To clarify the role of the myeloid lineage in regeneration competency, we investigated the myeloid cell populations present in regeneration-competent and -incompetent tadpole stages. To that end, we used our published cellular atlas generated by single cell mRNA sequencing from intact tail and amputation plane of regeneration-competent and -incompetent tadpoles at 1 dpa (Aztekin et al., 2019). Two uncharacterized myeloid cell clusters, myeloid 1 and myeloid 2, were present in both regeneration-competent and -incompetent tails. Myeloid 1 cluster was enriched for genes associated with inflammation (e.g. *tnf*) whereas myeloid 2 cluster was enriched for anti-inflammation (e.g. *arg1*) and reparative genes (e.g. *mmp1/9*) (Fig. 4A). Marker genes for these clusters were also enriched in peritoneal macrophages of adult frogs upon viral infection or bacterial stimulation (Fig. S7A-D). Based on these gene expressions, we classified these uncharacterized putative cell types as ‘inflammatory myeloid cells’ and ‘reparative myeloid cells’.

We observed a difference in the relative abundance of the inflammatory and reparative myeloid cells after amputation in regeneration-competent and -incompetent tadpoles. In intact tails, the ratio of inflammatory to reparative myeloid population was similar regardless of regeneration competency. However, at 1 dpa, regeneration-competent tadpoles had a dominant reparative myeloid cell population, whereas regeneration-incompetent tadpoles had a dominant inflammatory myeloid activity (Fig. 4B). Previously conducted bulk-RNA-seq following tail amputation detected reparative myeloid genes (e.g. *mmp7*, *g6pd*, *idh2*, *nampt*) as significantly upregulated (Love et al., 2011a), supporting the hypothesis that reparative myeloid cells might accumulate at the amputation plane to resolve inflammation levels in regeneration-competent tadpoles. Moreover, immune-suppressing drugs that can rescue the ‘no-regeneration’ phenotype (Fukazawa et al., 2009) also downregulate inflammatory myeloid gene expressions (Fig. 4C). Altogether, these observations indicate that the nature of the activated myeloid lineage differs between regeneration-competent and -incompetent tadpoles, and that regeneration incompetency might be associated with a failure to suppress an amputation-induced inflammatory state.

### Inhibition of the inflammatory response enables establishment of a regeneration-permissive environment in regeneration-incompetent tadpoles

**Fig. 5.**
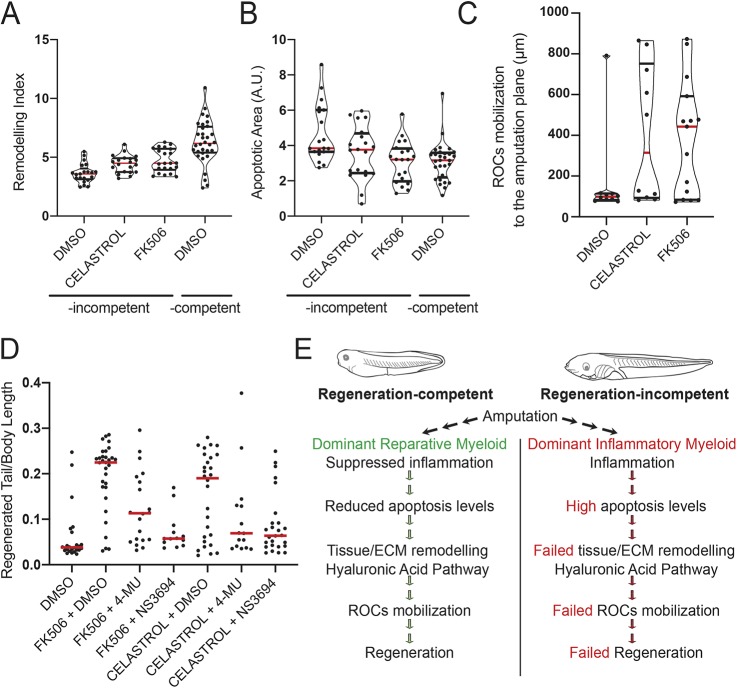
**Suppression of inflammation enables the emergence of a regeneration-permissive environment in regeneration-incompetent tadpoles.** (A) Remodelling index at 1 dpa in FK506- or Celastrol-treated regeneration-incompetent tadpoles. All samples were obtained from three biological replicates: DMSO *n*=22; FK506 *n*=21; Celastrol *n*=23. Red line denotes median, and black lines denote quartiles. Note that regeneration-competent 1 dpa DMSO data from Fig. 3B were re-plotted for comparison. DMSO versus Celastrol: *P*<0.05 (*t*-test), *P*<0.01 (Mann–Whitney *U*-test); DMSO versus FK506: *P*<0.01 (*t*-test), *P*<0.01 (Mann–Whitney *U*-test). (B) Apoptotic area levels at 1 dpa in FK506- or Celastrol-treated regeneration-incompetent tadpoles. All samples were obtained from three biological replicates: DMSO *n*=19; FK506 *n*=20; Celastrol *n*=20. Red line denotes median, and black lines denote quartiles. Note that regeneration-competent 1 dpa DMSO data from Fig. 3A were re-plotted for comparison. DMSO versus Celastrol: *P*<0.05 (*t*-test), *P*=0.065 (Mann–Whitney *U*-test); DMSO versus FK506: *P*<0.01 (*t*-test), *P*<0.01 (Mann–Whitney *U*-test). A.U., arbitrary unit. (C) ROC relocalization levels at 2 dpa in FK506- or Celastrol-treated regeneration-incompetent tadpoles. All samples were obtained from two biological replicates: DMSO *n*=10; FK506 *n*=15; Celastrol *n*=10. Red line denotes median, and black lines denote quartiles. (D) Immune-suppressing drug-mediated rescue of regeneration requires apoptosis and tissue remodelling. Regeneration-incompetent tadpoles were treated with FK506 or Celastrol either alone or in combination with an inhibitor of tissue remodelling (4-MU to block the HA pathway) or an inhibitor of apoptosis (NS3694). All samples were obtained from four biological replicates: FK506+DMSO *n*=33; FK506+4-MU *n*=20; FK506+NS3694 *n*=13; Celastrol+DMSO *n*=29; Celastrol+4-MU *n*=15; Celastrol+NS3694 *n*=28. Red line denotes median, each dot represents individual tadpoles. (E) Model of myeloid lineage involvement in regeneration-competent and -incompetent tadpoles. The myeloid lineage activity controls hierarchical cellular mechanisms that create the regeneration-permissive environment required for regeneration.

As our results suggest that a decrease in the abundance of inflammatory myeloid cells enables regeneration, we hypothesized that suppression of inflammation is required for the execution of the multiple cellular mechanisms required for regeneration. To test this, we investigated whether regeneration-incompetent tadpoles treated with immune-suppressing drugs mimic regeneration-competent tadpoles. In agreement with previous work (Fukazawa et al., 2009), treatment of regeneration-incompetent tadpoles with FK506 or Celastrol right after amputations restored the regenerative abilities of some but not all tadpoles (Fig. S8A,B). Furthermore, immune-suppressing drugs were able to reduce apoptosis levels and enable remodelling similar to regeneration-competent tadpoles (Fig. 5A,B). Moreover, ROC mobilization was also achieved in treated regeneration-incompetent tadpoles, although with a slight delay compared with regeneration-competent tadpoles in which ROCs relocalize within 24 h (Fig. 5C, Fig. S8C). In these early cellular mechanisms at 1 dpa, treatment with FK506 and Celastrol revealed two phenotypic populations, mirroring the ‘regenerated’ and ‘non-regenerated’ tadpoles phenotypes observed at 7 dpa.

Finally, blocking HA synthesis or apoptosis abrogated the rescue of regeneration mediated by immunosuppressive drugs (Fig. 5D, Fig. S8D) further confirming that suppression of inflammation is a key step to generating a regeneration-permissive environment. We conclude that the myeloid lineage, and its inflammatory state, controls apoptosis levels, which then enable remodelling of ECM and relocalization of ROCs to the amputation plane to ensure regeneration (Fig. 5E).

## DISCUSSION

In this article, we establish the essential role of myeloid lineage on tail regeneration by utilizing complementary depletion approaches. All three myeloid depletion strategies impaired regeneration efficiency, demonstrating the key role of the myeloid lineage in the process. Interestingly, when we injected control liposomes to tadpoles, we observed an increased inflammatory response, presumably occurring at the site of injection (trunk-ventral vein region). This increased activation of the myeloid lineage has very moderate impact on the regeneration potential compared with the effect of clodronate-mediated myeloid cell depletion. This suggests that the detrimental effect of an increased inflammatory phase requires the presence of activated myeloid cells at the amputation plane rather than the production of systemic factors. Our second approach applied genetic labelling to ablate *slurp1l* expressing myeloid cells. Although the *slurp1l* promoter is historically used to investigate myeloid lineage (Paredes et al., 2015; Smith et al., 2002), we found that this reporter is sensitive to amputation as exemplified by its activation in several additional cell types upon amputation. Moreover, in the *Xenopus* tail regeneration cell atlas, endogenous *slurp1l* was activated and/or upregulated in amputated tails in different cell types (e.g. ROCs, sclerotome, mesenchyme) (Aztekin et al., 2019). Hence, although this promoter seems suitable for tracing myeloid cell in intact tadpoles, additional markers are needed to trace myeloid cells in amputated animals.

In contrast to the need for myeloid cells in regeneration-competent tadpoles, immune cells were suggested to negatively affect the regenerative response in incompetent stages (Fukazawa et al., 2009). This prompted us to dissect differences in myeloid lineage of regeneration-competent and -incompetent tadpoles. By analysing the *Xenopus* tail cell atlas, we characterized two myeloid populations: inflammatory and reparative (Aztekin et al., 2019). Further work will be required to reveal if they are equivalent to mammalian polarized M1- and M2-like macrophages. Surprisingly, we did not record a cluster representing neutrophils in the atlas although they are suggested to be present in later-stage tadpoles (3 weeks old) (Paredes et al., 2015). It is unclear if this is because of a technical challenge of capturing neutrophils or if neutrophils develop in later stages. Importantly, we detected differential behaviour of myeloid populations in regeneration-competent and -incompetent tadpoles showing that the suppression of inflammation correlates with regeneration. Nonetheless, it remains uncertain whether inflammatory myeloid cells directly block regeneration. Identification of tissue-specific promoters for this population will enable the development of transplantation methods. Likewise, such promoters could be used to manipulate the behaviour of these cells, such as their proliferation, to test if their increased numbers can directly block regeneration.

Our results show that immunosuppression reduces amputation-induced apoptosis levels to rescue the ‘no-regeneration’ phenotype, but that blocking apoptosis altogether abrogates regeneration. These results further suggest that a certain amount of apoptosis is required for regeneration (Tseng et al., 2007). Further work will be required to understand how such a regulated level of apoptosis would mechanistically lead to successful regeneration. During *Hydra* head regeneration and zebrafish epithelial tissue maintenance, damage-induced apoptotic bodies were suggested to induce proliferation of cells (Brock et al., 2019; Chera et al., 2009) and this mechanism might be involved in *Xenopus* tail regeneration. Another unexplored possibility would be the recycling of components from dying cells to re-structure the regenerating tail.

In this work, we detected apoptosis using LysoSensor, which labels cells based on cytoplasmic acidity, a hallmark of apoptotic cells. We tested the specificity of this approach by comparing it with published caspase 3 immunohistochemistry analysis (Tseng et al., 2007), and tested it against an apoptosis inhibitor. As LysoSensor is sensitive to pH, we may have recorded high pH-containing cells other than apoptotic cells, including macrophages. However, as we detected more LysoSensor signal upon different myeloid lineage-depletion protocols and we did not detect this signal as sparse distinct cells, it is unlikely that it originates from recruited macrophages.

Previous bulk genomics approaches applied to amputation plane tissues during regeneration led to the hypothesis that tadpoles activate metabolic gene expression for regeneration (Love et al., 2011a; Love et al., 2014). Meanwhile, our results suggest that previously reported metabolic genes (e.g. *slc2a3*, *pkm*) are particularly enriched in the reparative myeloid lineage. Hence, it remains unclear whether the gene expression signature reported in previous work reflects a global metabolic change at the tissue/animal level or rather indicates a change of the myeloid cell status. This could happen through the attraction of reparative myeloid cells to the amputation plane, a switch from inflammatory to reparative states as seen in zebrafish (Nguyen-Chi et al., 2015), or both.

Although the role of the myeloid lineage is emphasized in different regeneration paradigms, the cellular mechanisms under its control are poorly defined. We found that myeloid lineage activity orchestrates a sequence of events encompassing the control of apoptosis level, the remodelling of the amputation plane, and the relocalization of ROCs. Our analysis enables us to position these essential cellular mechanisms with regard to each other in the sequence of events leading to regeneration. However, it does not show whether the relationship is direct. Indeed, although our proposed hierarchal cellular mechanism is one-directional, it is likely that reciprocal interactions exist between these cellular mechanisms. For example, ROCs also express metalloproteases (Aztekin et al., 2019), wound closure can affect histolysis in mice digit repair (Simkin et al., 2015), and HA is known to influence inflammation (Alibardi, 2017; Litwiniuk et al., 2016). Further work will be required to determine if these cellular mechanisms directly affect each other and if crosstalk mechanisms are indeed involved. Altogether, our results suggest that the myeloid lineage contributes to the establishment of a regeneration-permissive environment.

Understanding the inherent differences between regeneration-competent and -incompetent tadpoles will be crucial for modulating myeloid lineage activity. Hypoxia (Ferreira et al., 2018), hydrogen pump activity (Adams et al., 2007) and reactive oxygen species (Love et al., 2013; Ferreira et al., 2018) have been shown to be required for regeneration, and modulation of their activity can rescue the ‘no-regeneration’ phenotype. As these mechanisms are closely linked with attracting and influencing myeloid lineages (Chen et al., 2018; Tan et al., 2016; Varesio et al., 2016; Yoo et al., 2011), they may be potential upstream events impacting myeloid cell behaviour. Further work with transgenic lines enabling accurate myeloid cell tracking upon amputation will help to investigate these dynamic phenomena.

A conserved function of myeloid lineage activity might be related to specialized wound epidermis formation. First, depletion of macrophages during axolotl limb regeneration was shown to interfere with the formation of the specialized wound epidermis and expression of ROC marker genes (e.g. *Sp9*, *Msx2*, *Dlx3*) (Godwin et al., 2013). Similarly, depletion of macrophages was also suggested to impair histolysis and wound closure in mammalian digit tip repair (Simkin et al., 2017). Moreover, interfering with myeloid lineage limited patterning and proliferation in zebrafish fin regeneration (Petrie et al., 2014). Hence, characterization of cell types and cellular mechanisms in different species will be required to pinpoint the conserved role of the myeloid lineage.

The increased complexity of the immune system may contribute to an amplified inflammatory response, which would delay or limit the execution of the regenerative programme. Indeed, pre/post-metamorphosis amphibians with a more developed immune system show a reduced regenerative ability (Beck et al., 2009; King et al., 2012; Mescher et al., 2013; Monaghan et al., 2014). In our rescue experiments using inflammation-suppressing drugs, we also noted that ROC mobilization was postponed compared with that of regeneration-competent tadpoles. Moreover, repeated amputation of axolotl limb regeneration results in fibrosis reminiscent of a sustained inflammatory state (Bryant et al., 2017). In these scenarios, amputation-induced inflammation levels might be higher, delaying or preventing the creation of a regeneration-permissive environment.

Altogether, we utilized early *Xenopus* tadpoles, which have a developmentally limited type of immune cells, to reveal the essential role of myeloid lineage and inflammation in the emergence of a regeneration-permissive environment. Multiple studies have demonstrated that suppression of inflammatory myeloid cells can support repair in various mouse injury models ranging from spinal cord injury to hair regeneration, and reduce scar formation (Chu et al., 2019; Gensel et al., 2017). Hence, *Xenopus* might offer new avenues for drug and genetic screens to identify factors that can promote reparative myeloid lineage, possibly enabling new therapeutic approaches.

## MATERIALS AND METHODS

### Tadpole generation and husbandry

*Xenopus laevis* embryos and tadpoles were generated as previously described (Aztekin et al., 2019). *In vitro* fertilization was performed and embryos/tadpoles were maintained in 0.1× MMR [0.1 M NaCl, 2.0 mM KCl, 1 mM MgSO_4_, 2 mM CaCl_2_, 5 mM HEPES (pH 7.8)]. The Nieuwkoop and Faber (NF) developmental table was used for developmental staging (Nieuwkoop and Faber, 1994). Unless otherwise stated, wild-type *Xenopus* were used for experiments. Tadpole experiments were approved by the University Biomedical Services at University of Cambridge and complied with UK Home Office guidelines (Animal Act 1986). Outbred and J inbred frogs used for viral infection and bacterial stimulation were from the *X. laevis* research resource for immunology at the University of Rochester (https://www.urmc.rochester.edu/microbiology-immunology/research/xenopus-laevis.aspx) following standard husbandry methodology regularly updated by the *Xenopus* community (see http://www.xenbase.org/entry). All animals were handled in accordance with stringent laboratory and University Committee on Animal Research regulations (Approval number 100577/2003–151).

### Regeneration assays

Regeneration assays were performed as described before with minor modifications (Aztekin et al., 2019). Briefly, tail amputations were carried out by removing ∼30-50% of the tail using a sterile scalpel in regeneration-competent tadpoles (NF stage 40-41), and regeneration-incompetent tadpoles (NF stage 46-47). At 7 dpa, tadpoles were classified as follows: excellent (regenerated tail elongation was indistinguishable from normal tails, except for missing somite segmentation), good (regenerated tails had either a defect with elongation, or were missing fin regeneration), partial (regenerated tails were much shorter, or had defects in patterning with missing fin regeneration, or showed an elongated bulge formation), none (either a blunt end or a small stump was seen at the amputation plane). Regeneration index was calculated by multiplying the tadpole numbers showing excellent, good, partial or none regeneration phenotypes by 3, 2, 1 or 0 points, respectively. Then, this value was divided by the total number of tadpoles. Quantitative assessment of regeneration was carried out by fixing tadpoles at 7 dpa and imaging individual tadpoles with a stereomicroscope. The regenerated tail length to the body length ratio was measured by Fiji and was used as an indication of the extent of regeneration. Regenerated tail lacks somite segmentation, and this information was used to locate the amputation site. For tadpoles that were not able to regrow any tail, the stump length was measured. Investigators were not blinded during data acquisition or analysis. Any sample with fewer than three tadpoles in a replicate were omitted from further analysis. Any experiment with visible contamination or unexplained tadpole death was omitted from further analysis. A maximum of ten tadpoles were cultured in each well of a 6-well plate. Samples that had a problem during the mounting procedure were also excluded from further analysis. No statistical test was applied to pre-determine the sample sizes. Each experiment was performed with multiple biological replicates, with each batch derived from different male or female individuals.

### RNA isolation and RT-qPCR

Tadpole tails were collected at indicated times. Eight to ten tails were transferred into 350 µl of RNeasy Kit Lysis Buffer (Qiagen) and vortexed at 4°C for 2-3 min. Afterwards, samples were placed at −80°C° for a couple of hours or overnight. RNA was extracted via RNeasy Kit (Qiagen) according to the manufacturer's instructions. cDNA synthesis and RT-qPCR were carried out as previously described (Jullien et al., 2017). qPCR primers are listed in Table S1. All qPCR results were analysed by the 2^−ΔΔCT^ method. Gene expression values were first normalized to the values of housekeeping genes *ef1α* (*eef1a2*) and *gapdh* in experiments involving tadpoles, and adult frogs, respectively. Subsequently, values were normalized to a selected experimental condition.

### Liposome-based myeloid lineage removal

Liposomes (4.6 nl) containing PBS (Encapsome, 23 mg/ml, Encapsula Nano Sciences) or clodronate (Clodrosome, 23 mg/ml, Encapsula Nano Sciences) were injected to the ventral vein region of NF developmental stage 35 and NF stage 40 tadpoles (approximately 31 and 7 h before tail amputation). We did not co-inject with a dye to trace successfully injected embryos, and all samples that went through this protocol were included in further experiments and analysis. Assessment of the myeloid lineage removal was carried out by injecting Encapsome or Clodrosome to the *slurp1l*:*EGFP* transgenic line (Smith et al., 2002), and checking the EGFP signal in the vein regions at indicated time points. The area of veins was manually selected and the integrated density values were collected using Fiji. Additionally, tails were collected at the time of amputation to assess myeloid gene expression by RT-qPCR. Hence, the change in myeloid gene expression in RT-qPCR results was recorded for 7 h after the final injection. Encapsome and Clodrosome stocks were used within 2 months of first use.

### Genetic ablation of myeloid lineage

Nitroreductase (NTR)/Metronidazole (MTZ)-based genetic ablation of myeloid lineage was performed as previously described (Aztekin et al., 2019) except that a *slurp1l*:*NTR* construct was used. The *slurp1l*:*NTR* construct was cloned as described before (Love et al., 2011b). F_0_ transgenic lines were generated using I-SceI-mediated transgenesis as previously described (Ogino et al., 2006). Briefly, one-cell-stage embryos were injected with of a 3:1 mix of p4_Slurp1l:CFPNTR, and p4_*Slurp1l*:*VenusGFP* constructs with I-SceI enzyme (NEB, R0694L). After incubation at 14°C overnight or until neurula stages, embryos were switched to 23°C, then grown to the indicated stages. Transgenesis efficiency was assessed by evaluating on an epifluorescence stereomicroscope the VenusGFP fluorescence of injected tadpoles. Broad signal-emitting tadpoles were selected for the experiments. For regeneration assays, tadpoles were incubated in 10 mM MTZ (Sigma-Aldrich, M1547). MTZ was added 1 day before amputation, immediately after amputation, and 2 days post-amputation, then removed on 6 dpa. As MTZ is light sensitive, all samples were maintained in the dark in dishes covered with foil. Myeloid cell removal was assessed by RT-qPCR analysis of myeloid gene expression in the amputated tails. Hence, the change in myeloid gene expression in RT-qPCR results was recorded from samples that had been incubated in MTZ for 1 day.

### CRISPR/Cas9-based myeloid lineage removal

The first exon of *Spib.L* and *Spib.S* were sequenced and gRNAs were designed based on these results. gRNAs were produced as described (Bassett and Liu, 2014). To target *Spib.S* and *Spib.L* in the same tadpole, we mixed two gRNAs targeting these genes. gRNA targeting *Tyrosinase* (gTyr) was generated as previously described (Wang et al., 2015) and used as a control. Early one-cell-stage embryos were injected with 4.6 nl solution containing a total of 1 ng Cas9 protein (Invitrogen, A36498) and 1250-1500 pg gRNAs. Myeloid cell removal was assessed by RT-qPCR analysis of myeloid gene expression in amputated and in regenerated tails. The efficiency of gRNA cutting was assessed by TIDE analysis (Brinkman et al., 2014). Briefly, eight to ten tadpole tails were collected at the time of amputation and used to isolate genomic DNA using a DNeasy Blood & Tissue Kit (Qiagen) according to manufacturer instructions. The gRNA target regions were amplified via PCR and sequenced. Sequencing results were used to evaluate the insertion/deletion levels by TIDE software. Briefly, sequencing results of gTyr-injected samples were compared with sequencing results of gSpib-injected samples to determine the putative insertion deletion levels. Guide RNA sequences and sequencing primers are listed in Table S2.

### Immunofluorescence and imaging

Immunofluorescence and imaging experiments were carried out as previously described (Aztekin et al., 2019). Images in Fig. S2E were taken on a Leica SP8 confocal microscope with a 10×/0.4 HC PL Apo CS2 Air objective. The following laser lines were used: 405 nm (DAPI), 470-670 nm White Laser (Alexa Fluor 488, Alexa Fluor 594). Fiji was used for maximum projection of *z*-stacks and to adjust contrast to highlight biological relevance. All other images were taken on a Leica stereomicroscope with a microscope camera DFC7000T. If needed, images were cropped, flipped, and/or rotated to highlight biological relevance. Primary antibodies used were: GFP (Abcam, ab13970, 1:500), TP63 (clone 4A4) (Abcam, ab735, 1:200). Secondary antibodies used were: goat anti-chicken IgY (H+L) secondary antibody, Alexa Fluor 488 (Invitrogen, A11039, 1:500), goat anti-mouse IgG (H+L) cross-adsorbed ReadyProbes secondary antibody, Alexa Fluor 594 (Invitrogen, R37121, 1:500).

### Drug treatments

Tail amputations were carried out as described above and tadpoles were transferred to drug-containing wells immediately after tail amputations. Tadpoles were randomly allocated to drug-containing wells. Drugs were used with following final concentrations unless otherwise stated: 40 µM NS3694 (Sigma-Aldrich, N7787); 100 µM 4-MU (Sigma-Aldrich, M1381); 4.5 µM FK506 (Sigma-Aldrich, F4679); and 50 ng/ml Celastrol (Sigma-Aldrich, C0869). In all experiments, control samples had the same DMSO concentration as that in drug-treated samples (≤0.1% DMSO for single drug treatment and ≤0.2% DMSO in combined drug treatments).

### Apoptosis assay

LysoSensor (Invitrogen, L7535), LysoTracker (Invitrogen, L7526), Caspase 3/7 Green Detection Kit (Invitrogen, C10423) and MitoTracker (Invitrogen, M7512) were tested to detect apoptosis. Tail amputations were carried out as described above and tadpoles were transferred to 0.1× MMR containing 0.25-0.50 µM LysoSensor immediately after amputations. Samples were imaged at the indicated time points by a stereomicroscope without fixation. The area that had the highest signal to background ratio at the amputation plane was manually selected and quantified using Fiji.

### Remodelling index scoring

Tadpoles at the indicated time points were imaged by a stereomicroscope. The ratio of the length of the whole amputation plane to the length of the exposed posterior somitic region was measured by Fiji, and calculated as the remodelling index. Examples and schematic description are presented in Fig. 2 and Fig. S1.

### ROC mobilization assay

ROC mobilization was assessed as indicated before (Aztekin et al., 2019) with minor modifications. pbin7Lef:GFP transgenic testes were used to generate tadpoles and used for perturbations that are listed above. Lef1-positive tadpoles were sorted prior to experiments. Tadpoles were fixed 12-16 h after amputation and stained with anti-EGFP antibody as described above. Stereoscope images were taken and the degree of ROC mobilization was quantified by measuring the distance of the leading Lef1-positive cells (either dorsal or ventral) to the notochord on the amputation plane.

### Single cell RNA-sequencing analysis

The single cell RNA-seq data from Aztekin et al. (2019) was used to generate myeloid lineage heat maps and to estimate abundance of myeloid cells before and after tail amputation in regeneration-competent and -incompetent tadpoles. Differential abundance analysis was carried out as previously described (Aztekin et al., 2019).

### Adult frog experiments

FV3 (*Iridoviridae*) was grown and heat-killed bacteria generated as previously described (Robert et al., 2014). Two-year-old adult frogs were intraperitoneally injected with either FV3 (1×10^6^ PFU) or heat-killed *Escherischia coli* (10^7^ bacteria) in a volume of 100 µl of amphibian PBS. Mpeg fluorescent peritoneal leukocytes were harvested by lavage (Grayfer, 2018), stained with biotinylated anti-*X. laevis*-specific MHC class II monoclonal antibody and sorted by flow cytometry as described by Edholm (2018).

### Statistical tests

Statistical tests applied in experiments are indicated in the figure legends. Two condition comparisons were assessed by unpaired two-tailed Student's *t*-test. Experiments involving more than two conditions were assessed by ordinary one-way ANOVA. All calculations were done in GraphPad.

## Supplementary Material

Supplementary information

Reviewer comments
