# Supplementary material for: The myeloid lineage is required for the emergence of a regeneration-permissive environment following Xenopus tail amputation
Source: Development. 2020 Feb 5;147(3):dev185496. doi: 10.1242/dev.185496 (PMC7033733; doi:10.1242/dev.185496)
Supplement: Supplementary information [file develop-147-185496-s1.pdf]

Supplementary Figures

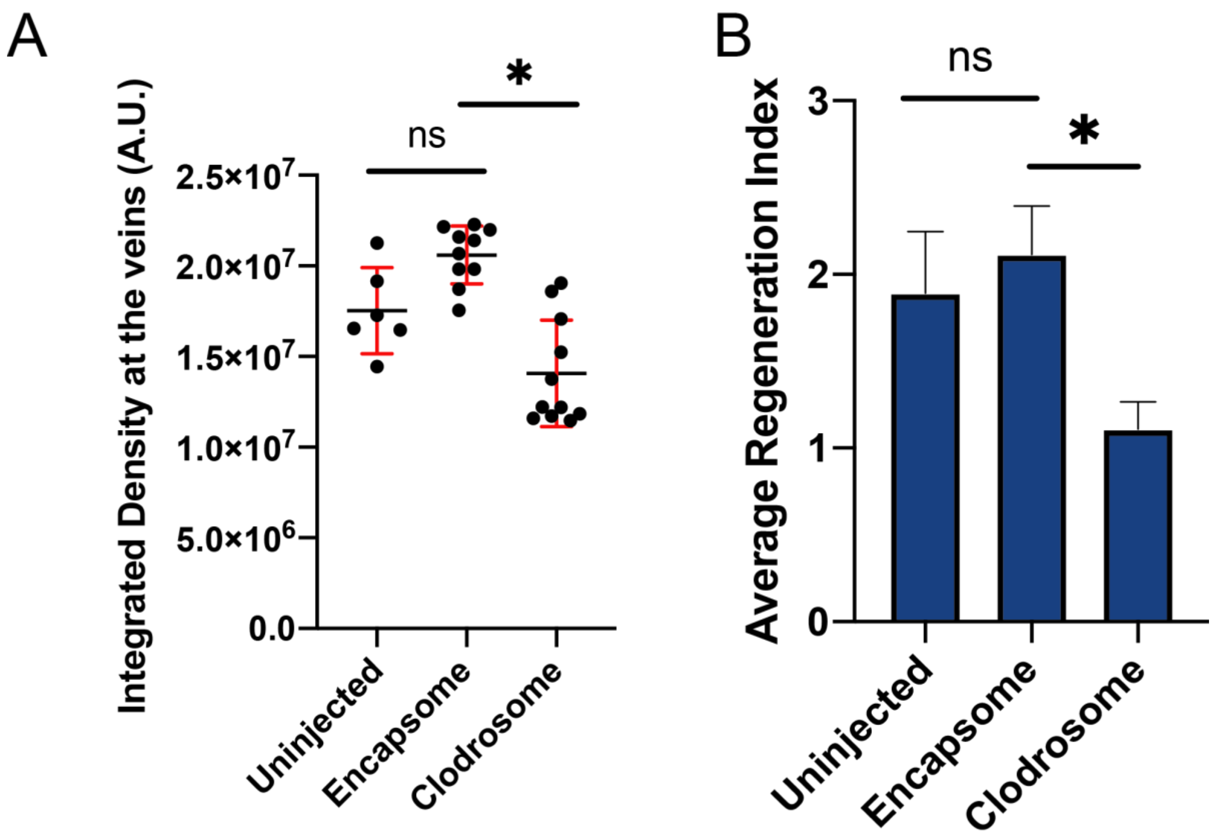

Fig S1. Clodrosome injection decreases myeloid lineage and inhibits regeneration. *(A)* Myeloid depletion efficiency following Encapsome or Clodrosome injections was assessed by quantifying the amount of *Slurp1l* expressing cell in the tail vein regions in regeneration-competent tadpoles 2 days after the last liposome injection. Student`s t-test was used to assess statistical significance; *$p<0.05$. *(B)* Average regeneration index in Uninjected, Encapsome, and Clodrosome injected tadpoles. *n* = 3 biological replicates for each sample, error bars represent ±s.e.m.. One-way ANOVA was used to assess statistical significance, *$p<0.05$. Note that this measurement was carried out on the same data as in Fig 1D.

Development • Supplementary information

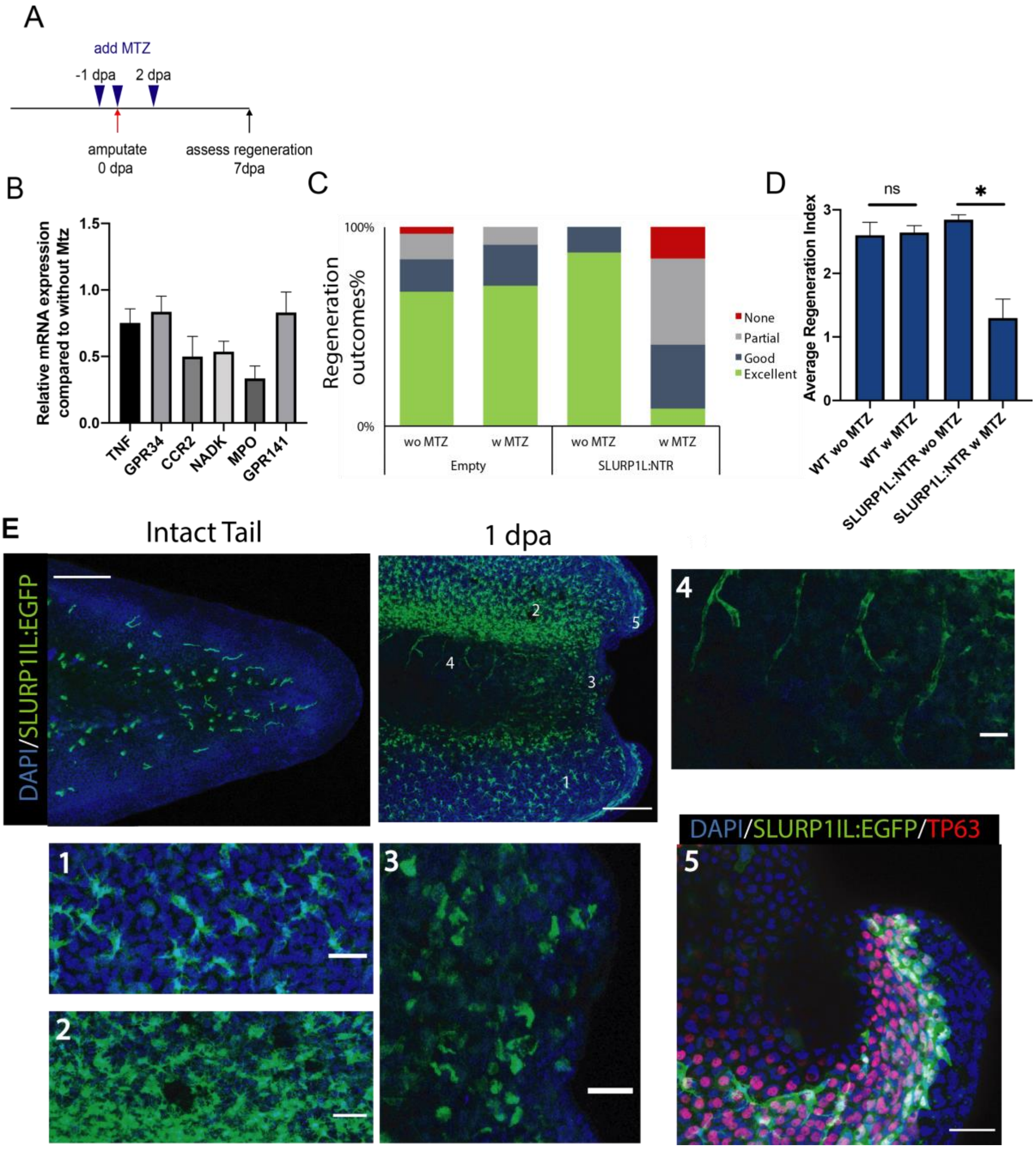

Fig S2. Genetic ablation of *Slurp1l* expressing cells decreases regeneration.

(A) Experimental design for ablating *Slurp1l* expressing myeloid cells using NTR/MTZ method. F0 transgenic tadpoles that express NTR under the control of *Slurp1l* promoter were generated. MTZ was added one day before, immediately after, and two-day after amputation in regeneration-competent tadpoles. Regeneration efficiency was assessed at 7 dpa. *(B)* NTR/MTZ mediated ablation of *Slurp1* expressing cells decreases myeloid cell lineage expression. Myeloid lineage gene expressions were assessed by RT-qPCR in *Slurp1l*:NTR expressing F0 tadpoles 1 day post MTZ treatment. Tail samples were collected at the time of amputation. All values were normalized to that of the controls without MTZ treatment. $n \geq 3$ biological replicates for each gene expression quantification, error bars represent ±s.e.m.. t-

Development • Supplementary information

test was used to assess statistical significance; **p<0.001.(C)* Regeneration-outcomes at 7 dpa following *Slurp1l* mediated NTR/MTZ myeloid cell ablation**.** All samples were obtained from three independent biological batches: Empty without (wo) MTZ *n* = 31; Empty with (w) MTZ *n* = 34; *Slurp1l*:NTR wo MTZ *n*= 47; *Slurp1l*:NTR w MTZ *n*= 44.*(D)* Average regeneration index at 7 dpa following *Slurp1l* mediated NTR/MTZ myeloid cell ablation. *n* = 3 biological replicates for each sample, error bars represent mean ±s.e.m. Student`s t-test was used to assess statistical significance, **p<0.05***.** Note that this measurement was carried out on the same data as in Fig 2C**.** *(E) Slurp1l* promoter gets activated upon amputation in non-myeloid lineage cells. (Left) In an intact tail Tg(*Slurp1l:*EGFP) transgenic line labels cells that resemble macrophages. (Middle) Upon tail amputation at 1 dpa Tg(*Slurp1l*:EGFP) signal is also seen in non-myeloid lineages. (Right) Examples of cells with different morphologies that are labelled by *Slurp1l* promoter. All samples were stained against EGFP (green), and DAPI (blue). Number 5 is also stained for TP63. ROCs reside at the edges of TP63 positive epidermal cells, and *Slurp1l* signal can also be seen in these cells. Scale bar= 250 µm for intact tail and 1 dpa image, 50 µm for zoomed images.

Development • Supplementary information

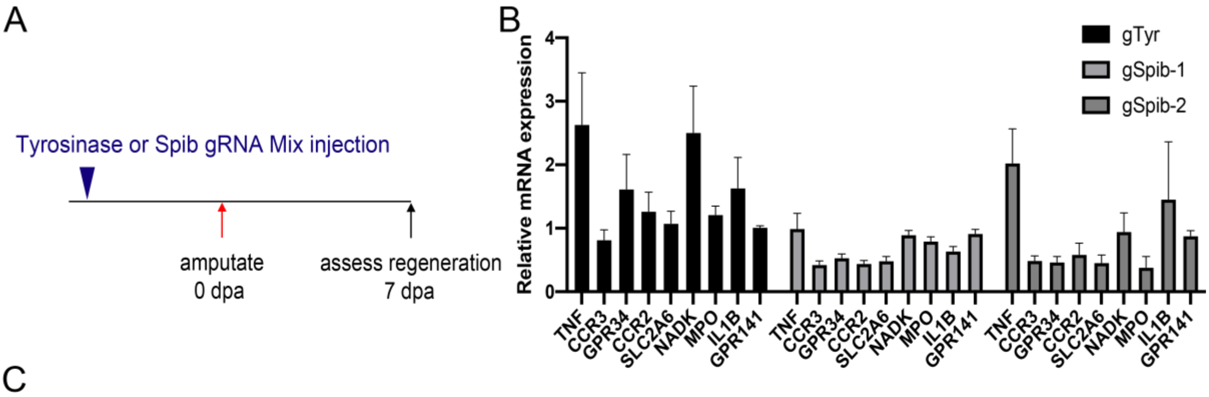

C

gSpib Mix target *Spib.L* and *Spib.S*

⬇

Collect NF 40 Tails for TIDE analysis

	Spib1.L	*Spib1.S*
gSpib Mix 1	71.40%	57.80%
gSpib Mix 2	64.80%	58.20%

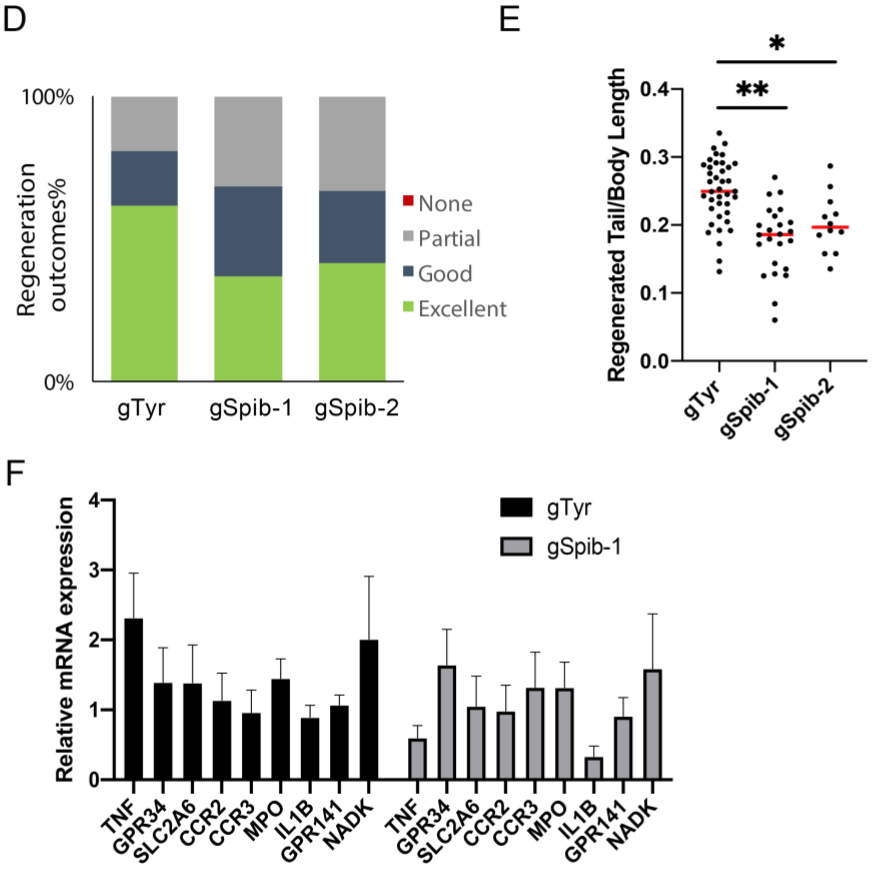

Fig S3. CRISPR/Cas9 mediated *Spib* knock-out decreases myeloid lineage gene expression and impairs regeneration.

(A) Experimental design for generating F0 tadpoles with a defective myeloid lineage *via* CRISPR/Cas9 mediated *Spib* knock-out and for testing regeneration in regeneration-competent tadpoles. Regeneration efficiency was assessed at 7 dpa. *(B)* Myeloid lineage gene expression was assessed by RT-qPCR in F0 tadpoles injected with guide RNAs targeting

Development • Supplementary information

either *Tyrosinase* (gTyr) as control, or *Spib* (gSpib). Spib-1 and Spib-2 denotes two different guide RNA mixes. Samples were collected at the time of tail amputation. All values were normalized to guide RNAs against *Tyrosinase* injected sample. Guide RNAs targeting *Spib* were able to decrease myeloid lineage gene expression. $n \geq 3$ biological replicates for each gene expression quantification, error bars represents ±s.e.m.. *(C)* Guide RNA efficiencies were assessed via TIDE analysis. At least 10 tails of tadpoles injected with gRNAs targeting *Tyrosinase* or *Spib* mixes were used for TIDE. *(D)* Regeneration-outcomes at 7 dpa for guide RNA injected tadpoles. *(E)* Regeneration was assessed in individual tadpoles by comparing regenerated tail to body length ratio at the 7 dpa. All samples were obtained from at least three independent biological batches: gTyr n = 39; gSpib-1 n= 24; gSpib-2 n= 11. One-way ANOVA used to assess statistical significance, red line denotes median, *$p<0.05$, **$p<0.001$. Note that this measurement was carried out on the same data as in Fig S3D. *(F)* Myeloid lineage gene expressions were assessed by RT-qPCR in F_0 tadpoles injected with guide RNAs targeting either *Tyrosinase* (as control), or *Spib1*. Spib-1 and Spib-2 denotes two different guideRNA mix. Samples were collected 7 day post tail amputation. All values were normalized to one sample of gTyr sample. GuideRNAs targeting *Spib* had no major difference in myeloid gene expression compared to gTyr control. n = 3 biological replicate for each gene expression quantification, and graphed, representing mean with ±s.e.m..

Development • Supplementary information

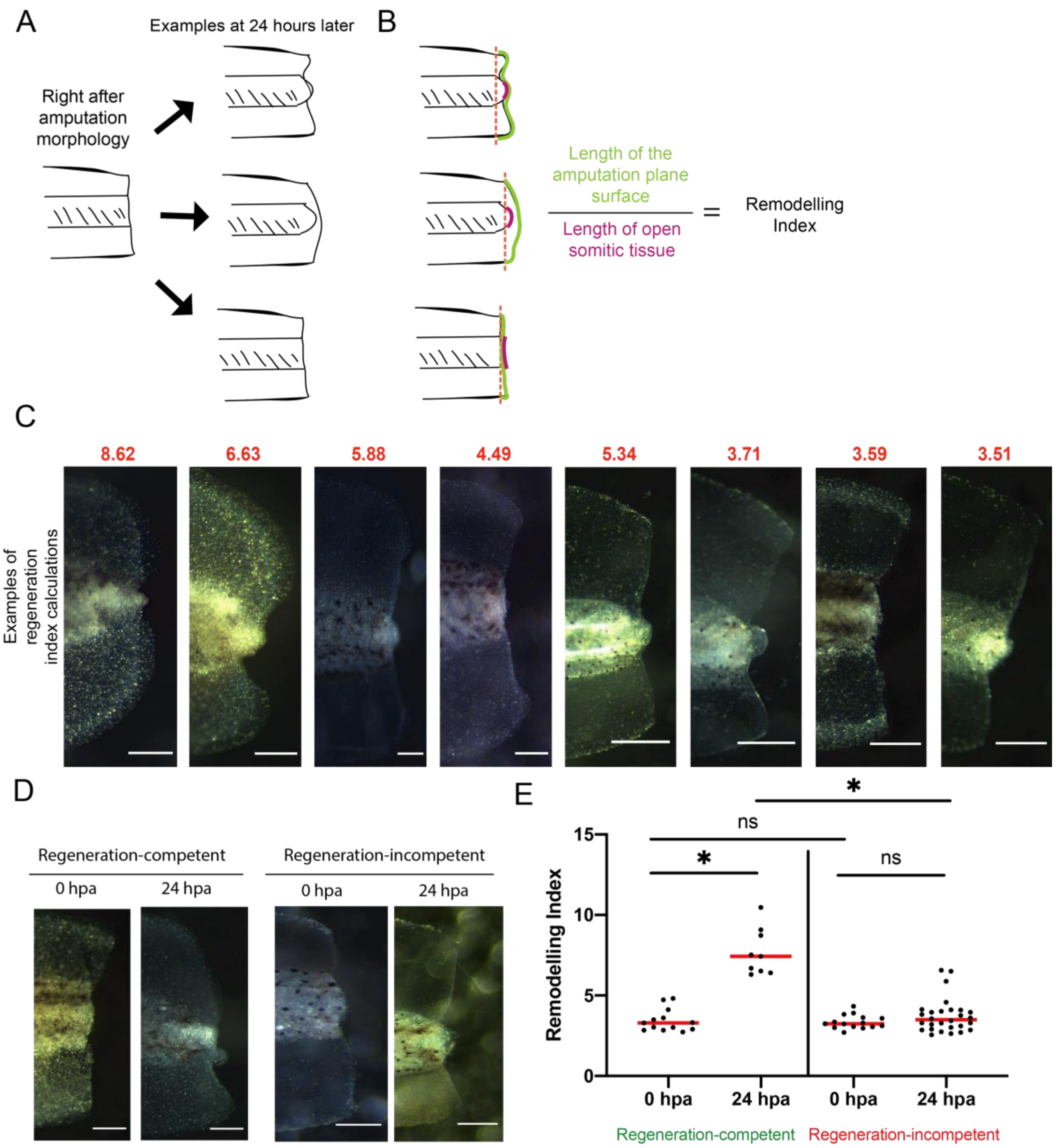

Fig S4. Remodelling index captures morphological differences between the amputation plane of regeneration-competent and -incompetent tadpoles.

(A) Schematics for amputation plane morphology immediately after amputation (left), and examples at 24 hours post amputation. Upon amputation, the somitic region is exposed to the outside medium and present a rectangular shape. Fin regions do not show any inward curvatures towards the amputation plane. Within 24 hours, somitic regions undergo histolysis and muscle tissue degeneration (Beck et al., 2003), while fin cells from the ventral and dorsal sides of the tail start covering the amputation plane. In regeneration-incompetent tadpoles, limited changes are observed (right, bottom). *(B)* Ratio of the length of the amputation plane surface to length of open somitic region used to estimate “remodelling index”. *(C)* Examples of images with corresponding regeneration index scores. *(D)* Representative images of

Development • Supplementary information

regeneration-competent and –incompetent tadpoles, 0-hour post amputation and 24 hours post amputations. Competent tadpole present extensive remodelling of the somatic and fin tissues. Scale bar= 250 µm. *(E)* Quantification of remodelling index for regeneration-competent and –incompetent 0 hpa, and 24 hpa samples. All samples were obtained from at least two biological replicates: Regeneration-competent 0 hpa *n*= 15; Regeneration-competent 24 hpa *n*= 9; Regeneration-incompetent 0 hpa *n*= 15; Regeneration-incompetent 24 hpa *n*= 28. One-way ANOVA used to assess statistical significance, red line denotes median, *$p<0.05$.

Development • Supplementary information

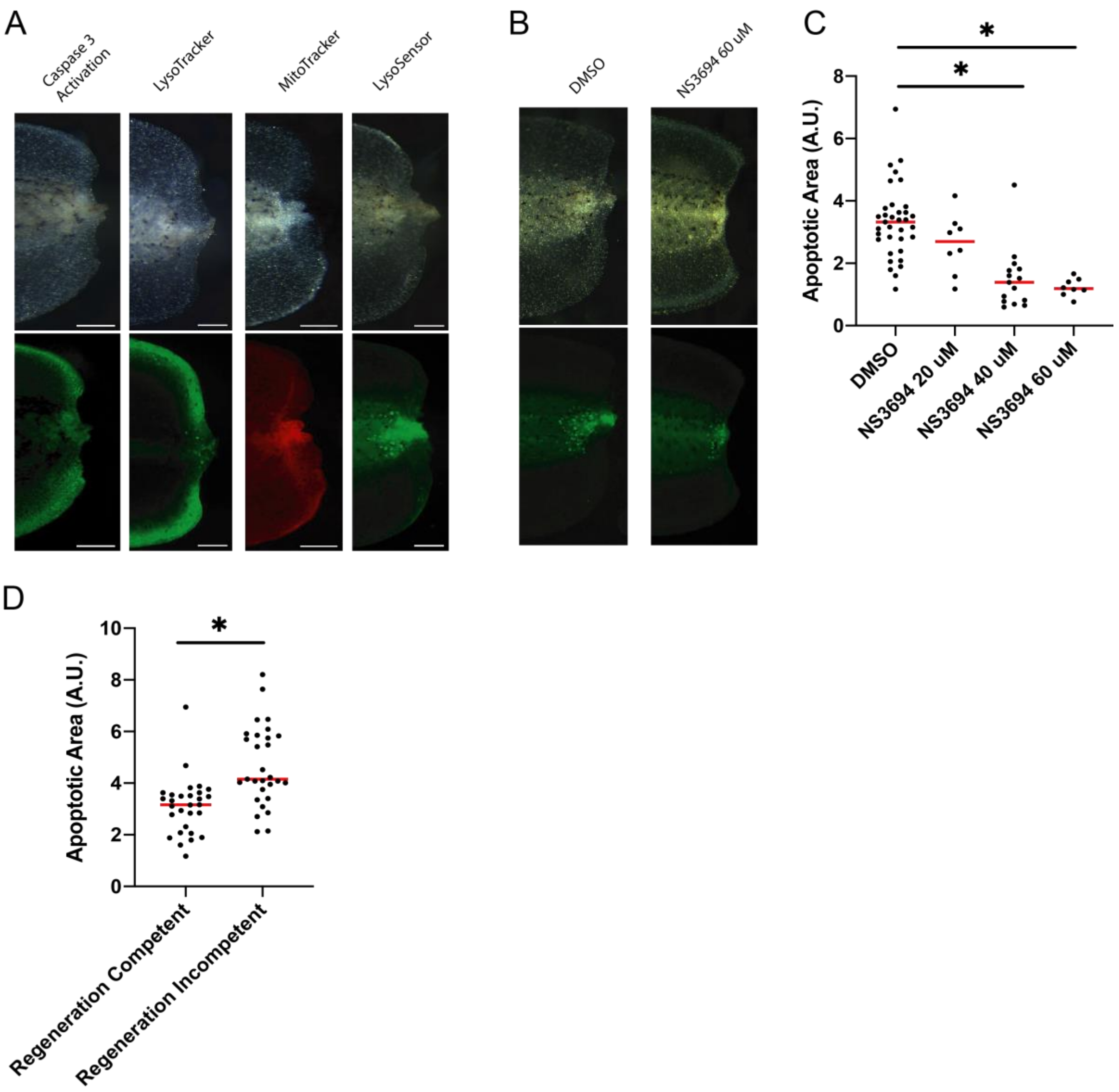

Fig S5. Lysosensor as an apoptosis reporter in amputated tail.

(A) Representative images for multiple reagents tested to detect apoptosis levels at 1 dpa in regeneration-competent tadpoles. Caspase 3/7 sensor and Lysotracker, two commonly used apoptosis detection methods present multiple off target signal in the tadpole fin region. Meanwhile, Mitotracker, and Lysosensor label more specifically the regions previously reported to have amputation induced apoptosis (Tseng et al., 2007). Scale bar= 250 µm. *(B)* Apoptosis inhibitor (NS3694, 60 µM) treatment decreases Lysosensor signal at the amputation plane. (C) Apoptosis area quantitation in control and 20, 40 ,60 µM NS3694 treated regeneration-competent tadpoles at 1 dpa. NS3694 treatments diminished the Lysosensor signal at 1 dpa. DMSO n = 35; 20 µM NS3694 n= 8; 40 µM NS3694 n= 15; 60 µM NS3694 n= 8. One-way ANOVA used to assess statistical significance, red line denotes median, *$p<0.05$. *(D)* Apoptotic area at 1 dpa in regeneration-competent and –incompetent tadpoles. Observed apoptosis levels are in agreement with the previously published reports

Development • Supplementary information

(Tseng et al., 2007). All samples were obtained from at least two biological replicates: Regeneration-competent *n* = 29; Regeneration-incompetent *n* = 28. Student`s t-test was used to assess statistical significance, red line denotes median, *$p<0.001$.

Development • Supplementary information

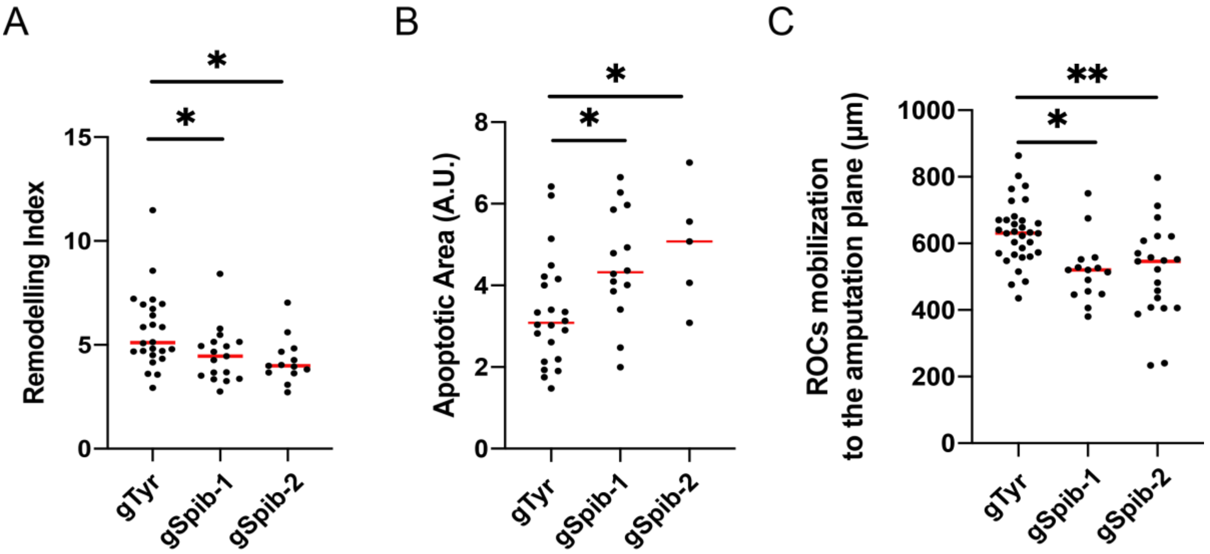

Fig S6. CRISPR/Cas9 *Spib* knock-out impairs relocalization of regeneration-organizing-cells, apoptosis levels, and tissue remodelling

(A) Quantification of ROCs relocalization at 1 day post amputation in regeneration-competent gTyr control and gSpib injected tadpoles*.* All samples were obtained from four independent biological batches: gTyr *n*= 39; gSpib-1 *n*= 37; gSpib-2 n=20. One-way ANOVA used to assess statistical significance, red line denotes median. **p<0.05, **p<0.001*. *(B)* Quantification of apoptosis levels at 1 day post amputation in regeneration-competent gTyr control and gSpib injected tadpoles. All samples were obtained at least from two independent biological batches except gSpib-2 was obtained from one batch: gTyr *n*= 21; gSpib-1 *n*= 14; gSpib-2 n=5. One-way ANOVA used to assess statistical significance, red line denotes median. **p<0.05*. *(C)* Quantification of remodelling index for the samples at 1 day post amputation in regeneration-competent gTyr control and *gSpib* injected tadpoles. All samples were obtained from two biological replicates: gTyr *n*= 24; gSpib-1 *n*= 17; gSpib-2 n=13. One-way ANOVA used to assess statistical significance, red line denotes median, **p<0.05.*

Development • Supplementary information

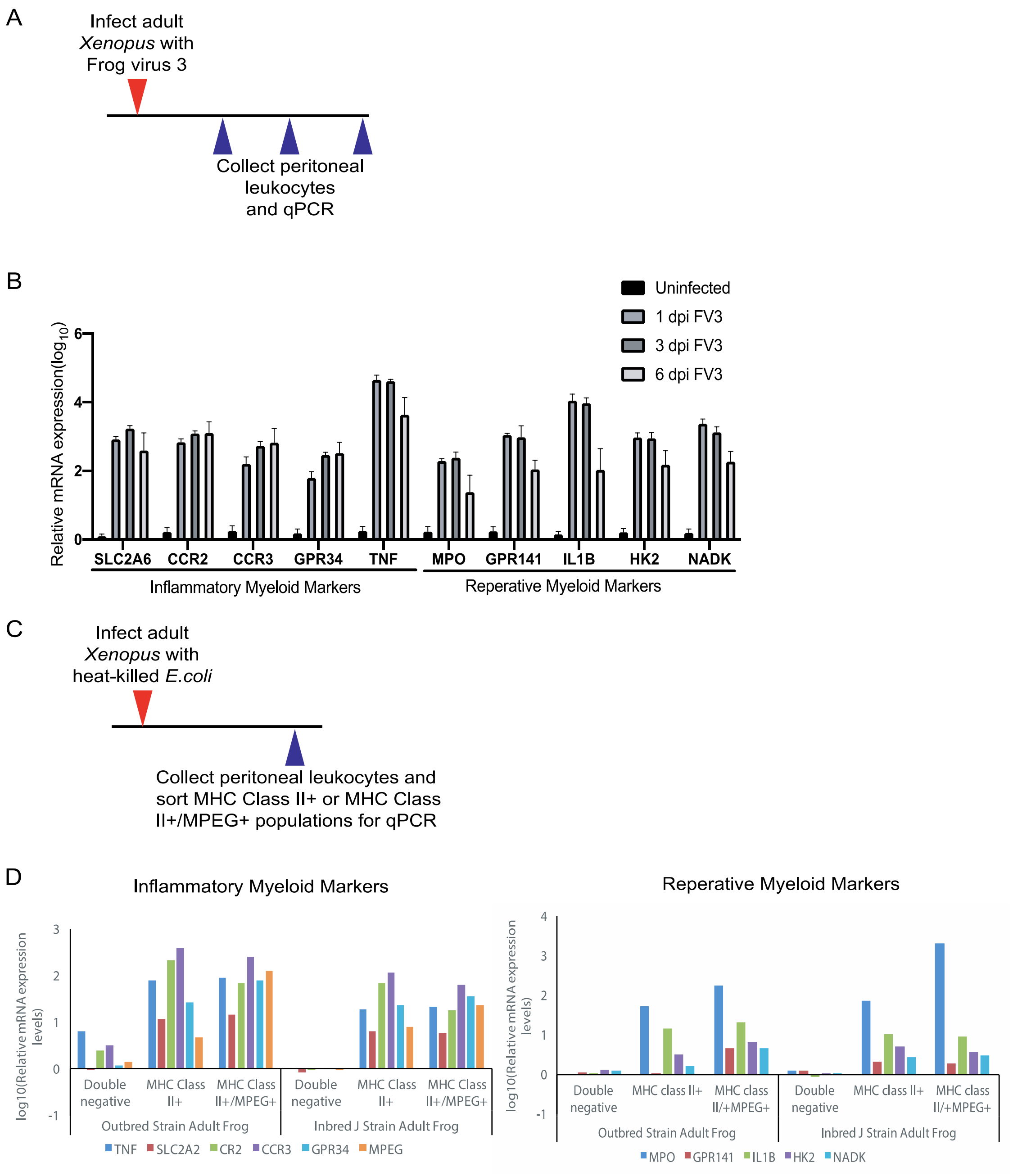

Fig S7. Adult *X. laevis* frogs infected with the ranavirus FV3 or stimulated with heat-shock killed bacteria upregulates inflammatory and reparative myeloid gene expression in peritoneal leukocytes.

(A) Experimental design for assessing the gene expression of different myeloid clusters upon Frog virus 3 infection (Large double strand DNA iridovirus). (*B*) Myeloid lineage gene expressions were assessed 1, 3, 6 days post-FV3 infection in peritoneal leukocytes. Inflammatory and reparative myeloid marker genes were enriched upon infection. *(C)* Experimental design for assessing the gene expression of different myeloid clusters of elicited peritoneal macrophages upon heat-killed *E. coli* stimulation. MHC Class II+, MHC Class II+/MPEG+, or double negative populations were sorted by flow cytometry from peritoneal

Development • Supplementary information

leukocytes and tested for myeloid gene expressions. (*D*) Myeloid lineage gene expressions were assessed 3 days post-elicitation of in peritoneal macrophages by heat-killed *E. coli*. (Left) Myeloid 1 and (Right) Myeloid 2 cluster genes are enriched upon infection.

Development • Supplementary information

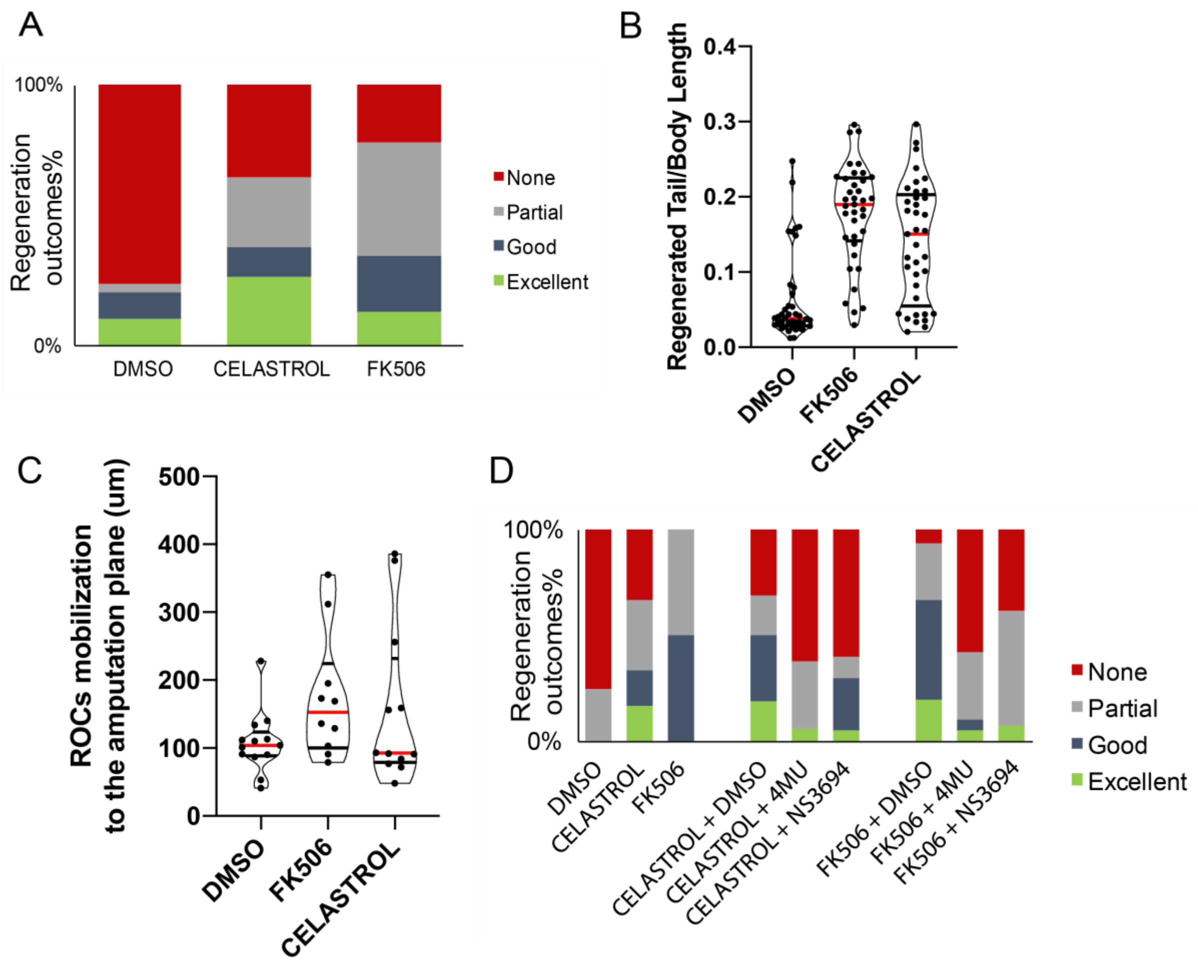

Fig S8. Immune suppressing drugs FK506 and Celastrol rescue the no-regeneration phenotype.

(A) Regeneration-outcomes at 7 dpa in FK506, or Celastrol treated tadpoles in regeneration-incompetent tadpoles. All samples were obtained from five biological replicates: DMSO *n* = 40; Celastrol *n*= 37; FK506 *n*= 37. *(B)* Regeneration was assessed in individual tadpoles by comparing regenerated tail to body length ratio at 7 dpa. Note that this measurement was carried out on the same data as in Fig 8A. *(C)* ROCs mobilisation initiates at 1 dpa in immune suppressing drugs treated regeneration-incompetent tadpoles. Quantification of ROCs relocalization at 1 day post amputation. All samples were obtained from two biological replicates: DMSO *n* = 13; FK506 *n*= 10; Celastrol *n*= 12. Red bar denotes the mean. *(D)* Immune suppressing drugs mediated rescue of regeneration requires apoptosis and tissue remodelling. Regeneration-outcome at 7 dpa in regeneration-incompetent tadpoles treated with FK506 or Celastrol either alone or in combination with inhibitor of tissue remodelling (4-MU to block HA pathway) or inhibitor of apoptosis (NS3694). Note that this measurement was carried out on the same data as in Fig 5D.

Development • Supplementary information

qPCR Primers

	Forward Primer	Reverse
gpr34.L	GACACAGACAAGCCCTCCAA	CCGCTAATGCAGTCCCAAGA
ccr2.L	AGCAGCTAAATTCCTTGTACCG	TCTGTCATGTTTCTGAGCTTTTTGT
ccr3.L	TGAACCTGAAATTTACATTTGCCCA	ACCCAGCAAACTGAAGGTGA
slc2a6.L	CACGTTTGGTTACGCCTTGG	AACACTGCCCCAAACCATGA
tnf.L	ATCCTCTTTGCTCTGCTGCAT	TGCTTGGGCTACTGGTACAGA
gpr141.L	ACGAATACACGAACGCTGACA	GCCATTCCCTGCGAACATAG
nadk.S	CCTGTGACTACGAGGAGGACT	TGAATTCCTTGGTGCTCCCTG
mpo.S	AAAAGCTGGTGGATACTGCGT	CTGGTTGCTTGAAGAACGCC
hk2.S	AGGGGCCTAATGAAAGCACC	GCCCCTCCAAGATCTAACACC
il1b.S	CCTGACTTGAGTTCCATTCCCA	AGCAGCATCTTCCATGTCGT
ef1a.S	GGAACGGTGACAACATGC	AGGCAGACGGAGAGGCTTA

Table S1: qPCR primer sequences
The forward and reverse qPCR primers sequences are indicated for each gene. Large and short chromosome information is also indicated in this list. These genes were picked based on their expression in inflammatory or reparative myeloid clusters in the *Xenopus* tail regeneration atlas (Aztekin et al., 2019).

guideRNA Sequences

**gSpib Mix #1**	
Spib.L-1	GGATTCACTCCGGTAAGTGA
Spib.S-1	GTCCACCCTGATCATGTTGT
**gSpib Mix #2**	
Spib.L-2	TGCTCTCAAAATGCTCAGCC
Spib.S-2	CCCTGATCATGTTGTGGGCA

TIDE Primers

	Forward Primer	Reverse Primer
Spib.L	AAAGTACAATGTGCCGTGCC	TCCCCTAATACCCAGCGACT
Spib.S	TGCACACAAGCTAGAATGCCT	GTGCAAGGTAAGCACGCTAT

Table S2: Guide RNA target and TIDE primer sequences
Guide RNA target sequences and TIDE primer sequences are indicated. TIDE primers were used for both validating the guide RNA target region and assessing insertion/deletion mutations due to gRNA injections.

Development • Supplementary information
