# Supplementary material for: The myeloid lineage is required for the emergence of a regeneration-permissive environment following Xenopus tail amputation
Source: Development. 2020 Feb 5;147(3):dev185496. doi: 10.1242/dev.185496 (PMC7033733; doi:10.1242/dev.185496)
Supplement: Reviewer comments [file develop-147-185496_review_history.pdf]

Development

The myeloid lineage is required for the emergence of a regeneration-permissive environment following *Xenopus* tail amputation

Can Aztekin, Tom W. Hiscock, Richard Butler, Francisco De Jesús Andino, Jacques Robert, John B. Gurdon and Jerome Jullien
DOI: 10.1242/dev.185496

Editor: Patrick Tam

Review timeline
Original submission: 9 October 2019
Editorial decision: 29 November 2019
First revision received: 21 December 2019
Accepted: 13 January 2020

Original submission

First decision letter

MS ID#: DEVELOP/2019/185496

MS TITLE: The myeloid lineage is required for the emergence of a regeneration permissive environment following Xenopus tail amputation

AUTHORS: Jerome Jullien, Can Aztekin, Tom Hiscock, Richard Butler, Francisco De Jesus Andino, Jacques Robert, and John Gurdon

I have now received all the referees' reports on the above manuscript, and have reached a decision. The referees' comments are appended below, or you can access them online: please go to BenchPress and click on the 'Manuscripts with Decisions' queue in the Author Area.

As you will see, the referees express considerable interest in your work, but have some significant criticisms and recommend a substantial revision of your manuscript before we can consider publication. If you are able to revise the manuscript along the lines suggested, which may involve further experiments, I will be happy receive a revised version of the manuscript. Your revised paper will be re-reviewed by one or more of the original referees, and acceptance of your manuscript will depend on your addressing satisfactorily the reviewers' major concerns. Please also note that Development will normally permit only one round of major revision.

Please attend to all of the reviewers' comments and ensure that you clearly highlight all changes made in the revised manuscript. Please avoid using 'Tracked changes' in Word files as these are lost in PDF conversion. I should be grateful if you would also provide a point-by-point response detailing how you have dealt with the points raised by the reviewers in the 'Response to Reviewers' box. If you do not agree with any of their criticisms or suggestions please explain clearly why this is so.

Reviewer 1

Advance summary and potential significance to field
Early Xenopus tadpoles are a well-established regeneration model, and exhibit different regenerative outcomes following partial tail amputation at different life stages. Here, the authors

© 2020. Published by The Company of Biologists under the terms of the Creative Commons Attribution License (https://creativecommons.org/licenses/by/4.0/).

first use a regeneration competent stage (stage 40/41), where the most likely outcome is a fully regenerated tail with some minor patterning defects such as a lack of somite segmentation. They show that the myeloid cell lineage is required for regeneration to proceed normally at this stage, using three complementary approaches. The results support the hypothesis, and add Xenopus tail regeneration to the list of vertebrate regenerative events requiring myeloid cells (normally macrophages), such as the spiny mouse and axolotl. Supporting this, they show that reduction of myeloid cells reduces apoptosis of the distal midline cells and prevented cells mobilising (the previously described "ROCs"). Excitingly, they reanalyse previous single cell data to define two myeloid cell lineages, which transcriptionally resemble the M1 and M2 macrophages described in mammals, in that one appears pro-inflammatory and the other reparative in function. These cells were found in different ratios in regeneration competent vs incompetent (stage 46/47) stage tadpoles suggesting that the presence of more inflammatory myeloid cells may result in failure of the tail to regenerate at these stages. To support this, they show that chemical suppression of the immune system rescues regeneration at incompetent stages, which suggests that modulation of the immune response is critical in establishing the correct conditions for regenerative responses. This nuance of pro and anti inflammatory response regulation has been long suspected, but not to my knowledge demonstrated at the cellular level in this model.
This paper therefore represents a significant and important step forward for the field and it will be interesting to see if older tadpoles use similar mechanisms to regenerate tails and limbs.

Comments for the author

I strongly recommend this manuscript for publication in Development. I have suggested minor revisions only. The study is robust and well presented and I commend the authors for providing alternate analyses of the same data in order to show both statistical analyses and - critically- variance.
1) Figure 1C the fluorescent cells are hard to see and perhaps adding a zoomed box would help?
2) DMSO is used as a vehicle for many of the drugs and I may have missed it but i do not think the % is stated anywhere. DMSO is a great solvent, but also anti inflammatory, and at 0.1% in our hands can alter regenerative outcome (negatively, stage 42/43) and so ideally a control group with no DMSO could have been included. Since the results are likely to be less striking compared to DMSO, I think simply stating the % would be sufficient. If DMSO was used at higher levels, this may have affected the results.
3) I would add macrophages to the keywords to help others find the work. I understand the use of "myeloid cells" is more appropriate, but it is more likely I think that most researchers would not think to use this term.
4) In several figures relating to regenerative outcomes, the statement "at least two biological replicates were used to obtain the data" is used. These appear to have been pooled. Given the variation in sib batches especially at refractory stages (40/41 is pretty robust), could these be coloured differently in the scatters to show any bias in/between sibling cohorts?

Reviewer 2

Advance summary and potential significance to field

The manuscript by Aztekin et al., aims to demonstrate that the myeloid lineage is essential for tail regeneration in the Xenopus laevis tadpole. There has been significant recent interest in the relationship between regenerative abilities in animals and the immune system. Altering the immune system in several animal models has established a relationship, but our understanding in the mechanisms regulating the relationship is still unclear. Therefore, the motivation behind this study is warranted and the study provides some new insights into how the immune system modulates regenerative abilities in animals. One difficulty in studying the interaction between two complex processes such as the immune system and regeneration is the interpretation of experiments and an ability to generate clear conclusions. Overall, I admire the fairness in the interpretation of the data, pointing out limitations and strengths of each experiment. Yet although this study provides a clear relationship between myeloid cells, apoptosis, and extracellular matrix, the interpretation is somewhat inflated when concluding the linear relationship between these events during regeneration.

© 2020. Published by The Company of Biologists under the terms of the Creative Commons Attribution License (https://creativecommons.org/licenses/by/4.0/).

Comments for the author

Although it is a strong manuscript, there are some flaws in the design of the study that limit the interpretation that can be made by the readers. These problems are described below.

1) To directly test the necessity of the myeloid lineage in regeneration, the authors injected regeneration-competent tadpoles with clodrosome containing liposomes, an agent that is supposed to specifically reduce the levels of myeloid cells. They show upon injection tadpoles are less likely to regenerate their tails. However, encapsome injection control also shows a significant decrease in regeneration. This raises the question that perhaps the act of depleting myeloid cells in the clodrosome group is only partially behind the reduced regeneration efficiency. Is it possible that immune cells increased when the encapsome was injected? If this is the case, and clodrosomes were not studied at all, the conclusion of the opening experiment would be that too many immune cells inhibit regeneration - the opposite conclusion to the study. Based upon Figure 1C, it should be possible to quantify whether immune cells increase with the encapsome rather than gene expression - “A drawback of this approach was the general upregulation of immune cell signal and inflammation which could potentially contribute to the phenotype.”

2) To support the claim that myeloid cells are needed for regeneration, the authors provide mRNA quantification of markers associated with the myeloid lineage in encapsome and clodrosome injected animals. Given the variability of regeneration outcome in figure 1d, it would be helpful to explain the phenotype of the animals used for mRNA quantification (i.e. an animal from the “excellent” category from the encapsome group is not being compared to an animal from the “none” category from the clodrosome group).

3) To further support the claim that the myeloid lineage is essential for regeneration, the authors generated tadpoles that express NTR under the control of the Slurp1 promoter. In these experiments, the authors see a decrease in the likelihood of tail regeneration. However Slurp1 expressing cells were observed in cells other than those within the myeloid lineage. The authors recognize this and state their observation of “Slurp1l promoter activation in non-myeloid lineages”. Given this, it is difficult to conclude definitively that depletion of the myeloid lineage is a causative factor to inhibit regeneration. Using the Spib1/2 promoter that was knocked out in the subsequent experiments may be a better driver gene. Including these experiments would strengthen the conclusion that myeloid cells specifically are necessary for regeneration.

4) The authors knock out Spib-1/Spib-2 genes, which are known markers for the myeloid lineage. In this experiment, the authors nicely show that KO of these genes reduces the regenerated tail/body length, although it us unclear why the previous metric (regeneration outcome %) was not again used as a measure of successful regeneration. These KO experiments are the most compelling data that support an essential role of the myeloid lineage during tail regeneration. One minor concern is that KO of these genes may produce an early lethality phenotype despite the animals being mosaic. This may raise the question that the reduced tail/body length observed in the KOs is due to an overall unhealthy animal instead of depletion of the myeloid lineage. A simple picture of the tadpoles at this stage would be enough to demonstrate a regeneration-specific phenotype.

5) It is difficult to make the connection between a decrease in possible remodeling and HA. The paragraph from line 122 to line 127 claims that because remodeling decreases after myeloid depletion, and HA is involved in ECM and remodeling, the myeloid lineage therefore controls HA deposition. The evidence for this direct link is lacking.

6) The authors state that apoptosis levels directly affect remodeling of ECM (198-199), but this is not tested in the manuscript.

7) Median is indicated in figures 1-4, but mean indicated in Figure 5.

8) In Figure 5, is there a regeneration-competent control for comparison? It is stated “immune-suppressing drugs were able to reduce apoptosis levels and enable remodeling similar to regeneration-competent tadpoles”. Please include this comparison in Figure 5a possibly from figure 3? Figure 5A and B do not show whether there is a significant difference compared to DMSO-treated animals.

© 2020. Published by The Company of Biologists under the terms of the Creative Commons Attribution License (https://creativecommons.org/licenses/by/4.0/).

In all, the authors show evidence that the myeloid lineage is directly required for tail regeneration in the tadpole, but the complexity of the system make a clear linear interpretation difficult. There are still significant conclusions, but Figure 5E indicates too linear of a model of regeneration based upon the experiments and results. It is highly likely that each of these phenotypes observed are not set in a linear fashion and likely have much more complex interactions than are depicted here.

Reviewer 3

Advance summary and potential significance to field

The advance made in this paper is small but useful for the community. It confirms several regulators of regeneration shown in other models and systems.

Comments for the author

The authors use several macrophage depletion strategies to confirm the essential role of macrophages in tissue regeneration already described in other systems. They compared macrophages at regeneration competent (stage 40-41) and incompetent tadpole stages (stage 46-47) and found divergent populations expressing some different markers. They then use an inhibitor of apoptosis and hyaluronic acid to show that ROC (wound epithelium) mobilization is affected and make the likely claim that myeloid cells are upstream. By using immunosuppressive drugs FK506 and Celastrol they show downregulation of both inflammatory myeloid cell genes and some reparative myeloid cell genes and make the leap to rescue regeneration in the regeneration in young incompetent tadpoles with some success pointing to “inflammatory macrophages” as the potential cause.

In general, it seems like most of the findings are already known in other systems. The authors do a great job at tying together several known myeloid related functions that will be helpful for some in the community.
The re-use of a single cell data set from their Science paper to identify changes in the myeloid phenotype does echo what is already known in several other systems (i.e. Zebrafish vs Medaka, mouse neonate vs adult) however this is a good contribution to the field from a greatly simplified semi-juvenile model. The approach of using immunosuppressant drugs to restore regenerative capacity in the larval xenopus during a regeneration incompetent phase has been done previously (Fukazawa et al 2009) and it is good to see that this paradigm holds true later in development. However, the authors have no evidence that these drugs are acting directly or solely on macrophages and so statements to the effect should be tempered somewhat.

Overall the paper is extremely well presented and well written. The individual experiments are of a high standard. Collectively they fail to prove that inflammatory macrophages are inhibitory. Direct evidence such as adoptive transfer of “inflammatory macrophages” into regeneration competent tissues, or inducible macrophage-proinflammatory gene overexpression, but these experiments may not be practical. It would be important to mention such weaknesses in the discussion.

Some other minor issues are listed below:
The authors state that “at 1 dpa regeneration-incompetent tadpoles have more apoptotic cells compared to regeneration-competent tadpoles (Fig. S5D).” In figure 2 the authors use clodrosomes to show enhanced apoptosis as measured by lysosensor. The authors then claim that “removal of the myeloid lineage from regeneration-competent tadpoles leads to increased apoptosis that the myeloid cells are involved in the regulation of apoptotic cell level, possibly through apoptotic cell clearance.. Hence, amputation induced apoptosis and its clearance via the myeloid lineage activity, is likely to be crucial for regeneration”

Following on from that, in Fig S5 they use lysosensor to detect apoptotic cells, and show decreases lysosensor detection at amputation plane with the apoptosis inhibitor (NS3694) However, in figure S8, treatment with the NS3694 inhibitor in combination with celastrol or FK506 makes regeneration worse. The conclusions made about the role of apoptosis seem inadequate and need further

© 2020. Published by The Company of Biologists under the terms of the Creative Commons Attribution License (https://creativecommons.org/licenses/by/4.0/).

commentary in particular why increased apoptosis is associated with regeneration incompetence, and/or a lack of macrophages reconciling with why inhibition of apoptosis is destructive for the rescue via immunosuppression.

Also, because Lysosensor can detect low pH lysosomes in macrophages it is unclear if they are measuring apoptotic cells or macrophage recruitment. This should be discussed.

Fig S2 would benefit from Inset boxes. Numbering in high magnification images are not clear.

First revision

Author response to reviewers' comments

Revision of manuscript MS ID#: DEVELOP/2019/185496 MS TITLE: The myeloid lineage is required for the emergence of a regeneration permissive environment following Xenopus tail amputation.

Please find below a point by point answer to reviewers' comments.

Reviewer 1
Advance summary and potential significance to field
Early Xenopus tadpoles are a well-established regeneration model, and exhibit different regenerative outcomes following partial tail amputation at different life stages. Here, the authors first use a regeneration competent stage (stage 40/41), where the most likely outcome is a fully regenerated tail with some minor patterning defects such as a lack of somite segmentation. They show that the myeloid cell lineage is required for regeneration to proceed normally at this stage, using three complementary approaches. The results support the hypothesis, and add Xenopus tail regeneration to the list of vertebrate regenerative events requiring myeloid cells (normally macrophages), such as the spiny mouse and axolotl. Supporting this, they show that reduction of myeloid cells reduces apoptosis of the distal midline cells and prevented cells mobilising (the previously described "ROCs"). Excitingly, they reanalyse previous single cell data to define two myeloid cell lineages, which transcriptionally resemble the M1 and M2 macrophages described in mammals, in that one appears pro-inflammatory and the other reparative in function. These cells were found in different ratios in regeneration competent vs incompetent (stage 46/47) stage tadpoles suggesting that the presence of more inflammatory myeloid cells may result in failure of the tail to regenerate at these stages. To support this, they show that chemical suppression of the immune system rescues regeneration at incompetent stages, which suggests that modulation of the immune response is critical in establishing the correct conditions for regenerative responses. This nuance of pro and anti inflammatory response regulation has been long suspected, but not to my knowledge demonstrated at the cellular level in this model.This paper therefore represents a significant and important step forward for the field and it will be interesting to see if older tadpoles use similar mechanisms to regenerate tails and limbs.

We thank the reviewer for his/her encouraging words. Particularly, we take note of the reviewer's acknowledgment that the concept of pro- and anti-inflammatory response affecting regeneration has long been suspected but was not evaluated at the cellular level in this model.

Reviewer 1 Comments for the author
I strongly recommend this manuscript for publication in Development. I have suggested minor revisions only. The study is robust and well presented and I commend the authors for providing alternate analyses of the same data in order to show both statistical analyses and - critically- variance.
1) Figure 1C the fluorescent cells are hard to see and perhaps adding a zoomed box would help?
In order to better show the location of GFP positive cells we have replaced the original images by zoomed in versions.

© 2020. Published by The Company of Biologists under the terms of the Creative Commons Attribution License (https://creativecommons.org/licenses/by/4.0/).

2) DMSO is used as a vehicle for many of the drugs and I may have missed it but i do not think the % is stated anywhere. DMSO is a great solvent, but also anti inflammatory, and at 0.1% in our hands can alter regenerative outcome (negatively, stage 42/43) and so ideally a control group with no DMSO could have been included. Since the results are likely to be less striking compared to DMSO, I think simply stating the % would be sufficient. If DMSO was used at higher levels, this may have affected the results.
We apologize for the incomplete description of the experimental setup. In our experiments, DMSO was diluted to either ≤0.1% (single drug) or ≤0.2% (double treatment). As the reviewer also points out, DMSO could also exhibit anti-inflammatory property, and we accounted for that possibility by setting DMSO controls with matching dilution (01/0.2%). We now include these information as part of the revised manuscript.

Page 14 line 435-437 : “In all experiments, control samples have the same DMSO concentration as that in drug treated samples (≤0.1% DMSO for single drug treatment and ≤0.2% DMSO in combined drug treatments).”

3) I would add macrophages to the keywords to help others find the work. I understand the use of "myeloid cells" is more appropriate, but it is more likely I think that most researchers would not think to use this term.
This is a very good suggestion and we have now added macrophages and neutrophils to the keywords list.

4) In several figures relating to regenerative outcomes, the statement "at least two biological replicates were used to obtain the data" is used. These appear to have been pooled. Given the variation in sib batches especially at refractory stages (40/41 is pretty robust), could these be coloured differently in the scatters to show any bias in/between sibling cohorts?

Regardless of regeneration competency, we didn’t observe any batch effect in the various regeneration assays used in the study. We believe that adding information related to batch origin will therefore complicate the figure unnecessarily. We show below an example of batch distribution at regeneration competent (Fig 3B) and one at the incompetent stage (Fig S8B). Please note however that at the regeneration incompetent stage the phenotypes seem to segregate into two populations. This is not depending on batch (see Fig S8B below) and is in an agreement with previously published results (Fukazawa et al., 2009).

Representative data for regeneration-incompetent tadpole experiment from Fig S8B. Each color represents a different biological replicate.

Representative data for regeneration-competent tadpole experiment from Fig 3B. Each color represents a different biological replicate.

Reviewer 2 Advance summary and potential significance to field
The manuscript by Aztekin et al., aims to demonstrate that the myeloid lineage is essential for tail regeneration in the Xenopus laevis tadpole. There has been significant recent interest in the relationship between regenerative abilities in animals and the immune system. Altering the immune system in several animal models has established a relationship, but our understanding in the mechanisms regulating the relationship is still unclear. Therefore, the motivation behind this study is warranted and the study provides some new insights into how the immune system modulates regenerative abilities in animals. One difficulty in studying the interaction between two complex processes such as the immune system and regeneration is the interpretation of experiments and an ability to generate clear conclusions. Overall, I admire the fairness in the interpretation of the data, pointing out limitations and strengths of each experiment. Yet, although this study provides a clear relationship between myeloid cells, apoptosis, and extracellular matrix, the interpretation is somewhat inflated when concluding the linear relationship between these events during regeneration.

We are sorry that the language came across as too definitive and thank the reviewer for pointing this issue. We completely agree that a direct connection between these cellular mechanisms is not established and that reciprocal interactions rather than a simple linear path is likely to be at play. In our work, we order these important cellular mechanisms without testing if there is a direct

© 2020. Published by The Company of Biologists under the terms of the Creative Commons Attribution License (https://creativecommons.org/licenses/by/4.0/).

relation between them. We could conclude that these cellular mechanisms operate upstream or downstream of each other. Their interaction being direct or indirect were not addressed in our work. In the discussion part of our submission, we commented on the possibility that the proposed linear model is likely to be more complex. Based on the reviewer comment and to avoid any confusion we now: (1) changed the proposed linear model figures (Fig 3 and Fig5) by replacing plain arrows with dashed arrows to indicate that the relationship between these cellular mechanisms could be indirect (2) extended the discussion section related to this point:
Previous version:
"While our proposed hierarchal cellular mechanism is one-directional, it is highly likely these cellular mechanisms also affect each other. For example, ROCs also express metalloproteases (Aztekin et al., 2019), wound closure can affect histolysis in mice digit repair (Simkin et al., 2015), and HA is known to influence inflammation (Alibardi, 2017; Litwiniuk et al., 2016)."

Current version:
"Our analysis enables us to position these essential cellular mechanisms with regard to each other in the sequence of events leading to regeneration. However, it does not show whether the relationship is direct. Indeed, while our proposed hierarchal cellular mechanism is one-directional, it is likely that reciprocal interactions exist between these cellular mechanisms. For example, ROCs also express metalloproteases (Aztekin et al., 2019), wound closure can affect histolysis in mice digit repair (Simkin et al., 2015), and HA is known to influence inflammation (Alibardi, 2017; Litwiniuk et al., 2016). Further work will be required to determine if these cellular mechanisms directly affect each other and if crosstalk mechanisms are indeed involved." Page 9-10 line 277-286

Reviewer 2 Comments for the author
Although it is a strong manuscript, there are some flaws in the design of the study that limit the interpretation that can be made by the readers. These problems are described below.

1) To directly test the necessity of the myeloid lineage in regeneration, the authors injected regeneration-competent tadpoles with clodrosome containing liposomes, an agent that is supposed to specifically reduce the levels of myeloid cells. They show upon injection, tadpoles are less likely to regenerate their tails. However, encapsome injection control also shows a significant decrease in regeneration. This raises the question that perhaps the act of depleting myeloid cells in the clodrosome group is only partially behind the reduced regeneration efficiency. Is it possible that immune cells increased when the encapsome was injected? If this is the case, and clodrosomes were not studied at all, the conclusion of the opening experiment would be that too many immune cells inhibit regeneration - the opposite conclusion to the study. Based upon Figure 1C, it should be possible to quantify whether immune cells increase with the encapsome rather than gene expression - "A drawback of this approach was the general upregulation of immune cell signal and inflammation which could potentially contribute to the phenotype."

We are not entirely sure we understand what the concern with Encapsome control is. Below we try to clarify the related observations and how they fit with our proposed model.

Any injection is likely to damage tissues and result in inflammation (also known as post-injection inflammation). Hence, an increase in immune cells and inflammation at the site of injection can be seen. In our study, tadpoles may have experienced such post-injection inflammation in the gut area, where the Encapsome/Clodrosome are delivered. However, when SLURP1L positive cells were quantified, the level of immune cell in the vein region of the tail (Fig S1A) do not show a statistically significant increase in Encapsosome injected tadpoles. Moreover, the reviewer comments that our Encapsome injection is significantly decreasing regeneration. However, this is not the case: when we quantify the average regeneration index for Uninjected and Encapsome injections, we do not see statistically significant difference (Fig S1B). The comparison between Encapsome and Clodrosome injection is used to reveal the effect of myeloid lineage depletion in otherwise identical experimental manipulations. When we compare Encapsome and Clodrosome injected tadpoles, we observe that Clodrosome injection is able to decrease immune cells, and the regeneration outcome. Hence, the side effects of injections do not interfere with the regeneration outcome, and the depletion of myeloid lineage seems to specifically block regeneration. Overall, these data do not contradict the conclusion from the study.

© 2020. Published by The Company of Biologists under the terms of the Creative Commons Attribution License (https://creativecommons.org/licenses/by/4.0/).

In light of the reviewer's comment we realise that the sentence concluding the manuscript Clodrosome section ("A drawback of this approach was the general upregulation of immune cell signal and inflammation which could potentially contribute to the phenotype.") might confuse the reader since it focuses on potential side effect rather than the main observation. Hence, we decided instead to conclude the section as follow:
Page 3 line 89-92 "…Moreover, upon tail amputations, these tadpoles had a reduced tail regeneration compared with control, Encapsome injected animals (Fig. 1D, Fig. S1B) , suggesting the myeloid lineage is required for regeneration. To independently assess the role of myeloid lineage in regeneration, we generated F0 transgenic tadpoles with a drug-inducible myeloid cell ablation construct in which the Nitroreductase (NTR) gene is under the control of the myeloid marker Slurp1l promoter."

We mention the potential drawbacks in our discussion:
Page 7 line 215-221 "Interestingly, when we injected control liposomes to tadpoles, we observed an increased inflammatory response, presumably occurring at the site of injection (trunk-ventral vein region). This increased activation of the myeloid lineage has very moderate impact on the regeneration potential when compared to the effect of Clodronate-mediated myeloid cell depletion. This suggest that the detrimental effect of an increased inflammatory phase requires the presence of activated myeloid cells at the amputation plane rather than the production of a systemic factors. "

2) To support the claim that myeloid cells are needed for regeneration, the authors provide mRNA quantification of markers associated with the myeloid lineage in encapsome and clodrosome injected animals. Given the variability of regeneration outcome in figure 1d, it would be helpful to explain the phenotype of the animals used for mRNA quantification (i.e. an animal from the "excellent" category from the encapsome group is not being compared to an animal from the "none" category from the clodrosome group).

The RT-qPCR analysis was carried out using pool of all the amputated tails within each experimental group. Hence, the values represent myeloid cells before regeneration even begin and there is no bias towards a particular regeneration outcome. Instead the RT-qPCR values reflect the overall expression pattern within the tails of tadpoles undergoing a given treatment. We provided this information in figure legends and methods in our original submission, and now included it in-text.
We have added a sentence to the figure legend to specify how RT-qPCR analysis was carried out.
Page 3 line 86
"…we observed reduced myeloid lineage cells and gene expressions at the time of tail amputation…"

Page 3 line 93
"When Metronidazole (MTZ) is added, NTR expressing cells are killed (Martinez-De Luna and Zuber, 2018) (Fig. S2A) as detected at the time of tail amputation."

Page 4 line 99-100
"Perturbing the Spib gene reduced the myeloid gene expressions at the time of tail amputation (Fig. S3A-C)."

3) To further support the claim that the myeloid lineage is essential for regeneration, the authors generated tadpoles that express NTR under the control of the Slurp1 promoter. In these experiments, the authors see a decrease in the likelihood of tail regeneration. However, Slurp1 expressing cells were observed in cells other than those within the myeloid lineage. The authors recognize this and state their observation of "Slurp1l promoter activation in non-myeloid lineages". Given this, it is difficult to conclude definitively that depletion of the myeloid lineage is a causative factor to inhibit regeneration. Using the Spib1/2 promoter that was knocked out in the subsequent experiments may be a better driver gene. Including these experiments would strengthen the conclusion that myeloid cells specifically are necessary for regeneration.
We appreciate the comments and agree with the reviewer that we cannot conclude that the myeloid lineage is required for regeneration based solely on the SLURP1L:NTR experiment results. Hence, we adapted two additional independent approaches that utilize different mechanisms of action and recognized by the literature. Encapsome/Clodrosome is the most specific and widely

© 2020. Published by The Company of Biologists under the terms of the Creative Commons Attribution License (https://creativecommons.org/licenses/by/4.0/).

used method to deplete macrophages and has been used to test regenerative outcome in many different species (Carrillo et al., 2016; de Preux Charles et al., n.d.; Godwin et al., 2017, 2013; Simkin et al., 2017). In our hands, it was also able to cause a significant decrease in the myeloid lineage population and lead to a strong loss of regeneration potency. That is why we deployed this assay in the remaining part of the manuscript as our main method.
Since we already successfully targeted Spib using CRISPR, we feel that interfering with Spib expressing cells using another method is unnecessary. Indeed, we already provide three completely independent ways of interfering with the myeloid lineage, all leading to the same findings and therefore already providing very strong support of our conclusion regarding the requirement of the myeloid lineage for regeneration.

4) The authors knock out Spib-1/Spib-2 genes, which are known markers for the myeloid lineage. In this experiment, the authors nicely show that KO of these genes reduces the regenerated tail/body length, although it us unclear why the previous metric (regeneration outcome %) was not again used as a measure of successful regeneration. These KO experiments are the most compelling data that support an essential role of the myeloid lineage during tail regeneration. One minor concern is that KO of these genes may produce an early lethality phenotype despite the animals being mosaic. This may raise the question that the reduced tail/body length observed in the KOs is due to an overall unhealthy animal instead of depletion of the myeloid lineage. A simple picture of the tadpoles at this stage would be enough to demonstrate a regeneration-specific phenotype.
We decided to perturb Spib gene as it has been reported by two independent groups to decrease myeloid lineage efficiently without negatively affecting early embryos survival. We further confirmed this by checking the viability of embryos at stage 22 and observed no lethality upon KO (see tables below). Moreover, it has been reported that if the myeloid lineage numbers are reduced but not ablated completely, with time the tadpoles can compensate myeloid lineage loss and remain healthy (Smith and Mohun, 2011). We also did not recognize any health problems or abnormality with tadpoles. Because of this, we did not record or take images of tadpoles at Stage 40. Nonetheless, we reanalyzed our existing tail images. Analysis of the distance between gut and tail tip length at the time of amputation (stage 40) show no significant difference between control and Spib KO embryos confirming a healthy tail development to that stage (see plot below).

Related to the metrics used for the Spib CRISPR experiments, we did provide the regeneration-outcome % in Fig S3D of our original submission. With this metric, we observed a reduction in the regeneration outcome albeit more modest than in our two other approaches. As this approach has milder effect, we further characterized the effect by assessing the length of regenerated tails. Our interpretation of this milder phenotype, in line with previous investigation using Spib morpholino (Costa et al., 2008; Smith and Mohun, 2011), is that our KO is mosaic and hence leading to partial depletion and eventual recovery of myeloid cell in later stage of development. Such recovery is supported by our observation that myeloid gene expressions are restored to control level at 7 dpa (Fig S2F).

Batch 1	Uninjected	gTyr	gSpib-1	gSpib-2
Injected Embryos	53	66	58	60
Viable at Stage 22	52	62	57	59
Viability%	98.1	93.9	98.3	98.3

Batch 2	Uninjected	gTyr	gSpib-1	gSpib-2
Injected Embryos	58	58	50	65
Viable at Stage 22	54	54	46	63
Viability%	93.1	93.1	92.0	96.9

5) It is difficult to make the connection between a decrease in possible remodeling and HA. The paragraph from line 122 to line 127 claims that because remodeling decreases after myeloid depletion, and HA is involved in ECM and remodeling, the myeloid lineage therefore controls HA deposition. The evidence for this direct link is lacking.
And
6) The authors state that apoptosis levels directly affect remodeling of ECM (198-199), but this is not tested in the manuscript.

© 2020. Published by The Company of Biologists under the terms of the Creative Commons Attribution License (https://creativecommons.org/licenses/by/4.0/).

In our initial submission, we did not state it is a direct effect but rather indicated “We conclude that the regulation of tissue remodeling by the myeloid lineage involves control of HA deposition.” Page 5 line 130-131.
Similarly, in our initial submission, we did not state that this effect is “direct” affect but rather proposed “…that the myeloid lineage, and its inflammatory state, controls apoptosis levels which then enable remodeling of ECM…”. Page 7 line 207-209.
To clarify this point and avoid any confusion, we have expanded on the discussion of our proposed linear model and potential direct and indirect effects:

“Our analysis enables us to position these essential cellular mechanisms with regard to each other in the sequence of events leading to regeneration. However, it does not show whether the relationship is direct. While our proposed hierarchal cellular mechanism is one-directional, it is likely that reciprocal interactions exist between these cellular mechanisms. For example, ROCs also express metalloproteases (Aztekin et al., 2019), wound closure can affect histolysis in mice digit repair (Simkin et al., 2015), and HA is known to influence inflammation (Alibardi, 2017; Litwiniuk et al., 2016). Further work will be required to determine if these cellular mechanisms directly affect each other and if crosstalk mechanisms are indeed involved.” Page 9-10 line 277-286

7) Median is indicated in figures 1-4, but mean indicated in Figure 5.
Thanks for pointing this. We should have explained why we choose to do this. In Figures1-4, our results have a normal distribution due to the expected variance and we think the median would serve as the best approximate to represent tadpoles in that group. Meanwhile, in agreement with previous publication (Fukazawa et al., 2009), not all tadpoles restore their regenerative abilities with the treatment of FK506 and Celastrol and we also do not see a normal distribution of phenotypes in Figure 5. Hence, the median would represent a tadpole state that is artificial. Due to this, we provided the mean as an estimate for approximate difference between conditions to highlight the potential effect. Nonetheless, for readers to distinguish this lack of normal distribution in phenotypes, we now present all the regeneration-incompetent rescue data with violin plots with individual data points including median and variance quartiles. Furthermore, we mention this data distribution and appearance of two phenotypes in-text.

Page 7 line 194-203
“In agreement with previous work (Fukazawa et al., 2009), treatment of regeneration-incompetent tadpoles via FK506 or Celastrol right after amputations restored regenerative abilities of some but not all tadpoles (Fig. S8A-B). Furthermore, immune-suppressing drugs were able to reduce apoptosis levels and enable remodelling similar to regeneration-competent tadpoles (Fig. 5A-B). Moreover, ROCs mobilization was also achieved in treated incompetent tadpoles, although with a slight delay when compared to regeneration-competent tadpoles in which ROCs relocalize within 24 hours (Fig. 5C, Fig. S8C). In these early cellular mechanisms at 1 dpa, treatments with FK506 and Celastrol majorly revealed two phenotypic populations, mirroring the “regenerated” and “non-regenerated” tadpoles phenotypes observed at 7 dpa. “

8) In Figure 5, is there a regeneration-competent control for comparison? It is stated “immune-suppressing drugs were able to reduce apoptosis levels and enable remodeling similar to regeneration-competent tadpoles”. Please include this comparison in Figure 5a, possibly from figure 3?
Thank for this suggestion that indeed enables a better assessment of the level to which regeneration is rescued. We included remodelling index and apoptosis levels of regeneration-competent tadpole 1 dpa samples treated with DMSO (from Fig 3) to Fig 5a and b.

9/ Figure 5A and B do not show whether there is a significant difference compared to DMSO-treated animals.
We hoped the data distribution based on scatter plots were clear enough to show the difference. To make it clearer, we now changed all data related to regeneration-incompetent rescue experiments to violin plots while keeping individual data points. When we conduct t-test or ANOVA to assess statistical significance, data in Fig 5a and b were statistically significant ($p<0.05$). However, in agreement with previous publication (Fukazawa et al., 2009), not all tadpoles restore their regenerative abilities with the treatment of FK506 and Celastrol and the data does not have a normal distribution (see also reviewer’s point 7 for additional details). As t-test and ANOVA may not

© 2020. Published by The Company of Biologists under the terms of the Creative Commons Attribution License (https://creativecommons.org/licenses/by/4.0/).

be the best approaches to test statistical significance for such data, we also conducted a Mann-Whitney U test which is more suitable for non-Gaussian distributions. When we apply this method, the comparison between DMSO and FK506 were statistically significant, but the comparison between DMSO and Celastrol for apoptosis levels were marginally not statistically significant with a p=0.065. We now present the data as violin plot to illustrate the lack of normal distribution and illustrate the phenotypes better. We also included the results of t-test and Mann Whittney U test in the figure legends.

"…Red line denotes median, and black lines denote quartiles. Note that regeneration-competent 1 dpa DMSO data from Fig 3B were replotted for comparison. DMSO vs Celastrol: $p<0.05$ (t-Test), $p<0.01$ (Mann Whittney U test), DMSO vs FK506: $p<0.01$ (t-Test), $p<0.01$ (Mann Whittney U test), (B) Apoptotic area levels at 1 dpa in FK506 or Celastrol treated regeneration-incompetent tadpoles. All samples were obtained from 3 biological replicates: DMSO n = 19; FK506 n= 20; Celastrol n= 20. Red line denotes median, and black lines denote quartiles. Note that regeneration-competent 1 dpa DMSO data from Fig 3A were replotted for comparison. DMSO vs Celastrol: $p<0.05$ (t-Test), $p=0.065$ (Mann Whittney U test), DMSO vs FK506: $p<0.01$ (t-Test), $p<0.01$ (Mann Whittney U test), (C) ROCs relocalization levels at 2 dpa in FK506 or Celastrol treated regeneration-incompetent tadpoles. All samples were obtained from two biological replicates: DMSO n = 10; FK506 n= 15; Celastrol n= 10. Red line denotes median, and black lines denote quartiles…"
Page 23-24 line 777-790

In all, the authors show evidence that the myeloid lineage is directly required for tail regeneration in the tadpole, but the complexity of the system make a clear linear interpretation difficult. There are still significant conclusions, but Figure 5E indicates too linear of a model of regeneration based upon the experiments and results. It is highly likely that each of these phenotypes observed are not set in a linear fashion and likely have much more complex interactions than are depicted here.

We thank again for pointing this out and hope our adjustment to text make it clear (please refer to our response to points 5&6).

Reviewer 3 Advance summary and potential significance to field
The advance made in this paper is small but useful for the community. It confirms several regulators of regeneration shown in other models and systems.

Reviewer 3 Comments for the author
The authors use several macrophage depletion strategies to confirm the essential role of macrophages in tissue regeneration already described in other systems. They compared macrophages at regeneration competent (stage 40-41) and incompetent tadpole stages (stage 46-47) and found divergent populations expressing some different markers. They then use an inhibitor of apoptosis and hyaluronic acid to show that ROC (wound epithelium) mobilization is affected and make the likely claim that myeloid cells are upstream. By using immunosuppressive drugs FK506 and Celastrol they show downregulation of both inflammatory myeloid cell genes and some reparative myeloid cell genes and make the leap to rescue regeneration in the regeneration in young incompetent tadpoles with some success pointing to "inflammatory macrophages" as the potential cause.

In general, it seems like most of the findings are already known in other systems. The authors do a great job at tying together several known myeloid related functions that will be helpful for some in the community.
The re-use of a single cell data set from their Science paper to identify changes in the myeloid phenotype does echo what is already known in several other systems (i.e. Zebrafish vs Medaka, mouse neonate vs adult) however this is a good contribution to the field from a greatly simplified semi-juvenile model. The approach of using immunosuppressant drugs to restore regenerative capacity in the larval xenopus during a regeneration incompetent phase has been done previously (Fukazawa et al 2009) and it is good to see that this paradigm holds true later in development. However, the authors have no evidence that these drugs are acting directly or solely on macrophages and so statements to the effect should be tempered somewhat.

Overall the paper is extremely well presented and well written. The individual experiments are of a high standard. Collectively they fail to prove that inflammatory macrophages are inhibitory. Direct

© 2020. Published by The Company of Biologists under the terms of the Creative Commons Attribution License (https://creativecommons.org/licenses/by/4.0/).

evidence such as adoptive transfer of “inflammatory macrophages” into regeneration competent tissues, or inducible macrophage-proinflammatory gene overexpression, but these experiments may not be practical. It would be important to mention such weaknesses in the discussion.

We thank the reviewer for the encouraging words and summary. We take note of the fact that there is agreement on the value of tying up known important cellular mechanisms to provide a more integrative framework of regeneration processes.

We agree with the reviewer that we do not provide direct evidence that “inflammatory macrophages block regenerative outcome”. We indeed only propose this as a possible mechanism but we did not test it directly as it is beyond the scope of this investigation. As suggested, we now comment on these limitations in the discussion section:

Page 8 line 241-246: “…Nonetheless, it remains uncertain whether inflammatory myeloid cells directly block regeneration. Identification of tissue-specific promoters for this population will enable developing transplantation methods. Likewise, such promoters can be used to manipulate the behaviour of these cells, such as their proliferation, to test if their increased numbers can directly block regeneration.”

Some other minor issues are listed below:
The authors state that “at 1 dpa regeneration-incompetent tadpoles have more apoptotic cells compared to regeneration-competent tadpoles (Fig. S5D).” In figure 2 the authors use clodrosomes to show enhanced apoptosis as measured by lysosensor. The authors then claim that “removal of the myeloid lineage from regeneration-competent tadpoles leads to increased apoptosis that the myeloid cells are involved in the regulation of apoptotic cell level, possibly through apoptotic cell clearance.. Hence, amputation induced apoptosis, and its clearance via the myeloid lineage activity, is likely to be crucial for regeneration”

Following on from that, in Fig S5 they use lysosensor to detect apoptotic cells, and show decreases lysosensor detection at amputation plane with the apoptosis inhibitor (NS3694) However, in figure S8, treatment with the NS3694 inhibitor in combination with celastrol or FK506 makes regeneration worse. The conclusions made about the role of apoptosis seem inadequate and need further commentary in particular why increased apoptosis is associated with regeneration incompetence, and/or a lack of macrophages reconciling with why inhibition of apoptosis is destructive for the rescue via immunosuppression.

We agree that the need for a controlled level of apoptosis to achieve regeneration is a bit perplexing and we therefore further discuss why this may be the case:

Page age 8-9 line 247-256
“Our results show that immunosuppression reduces amputation-induced apoptosis levels to rescue no-regeneration phenotype, but that blocking apoptosis altogether abrogates regeneration. These results further suggest that a certain amount of apoptosis is required for regeneration (Tseng et al., 2007). Further work will be required to understand how such regulated level of apoptosis would mechanistically leads to successful regeneration. During hydra head regeneration and zebrafish epithelial tissue maintenance, damage induced apoptotic bodies were suggested to induce proliferation of cells (Brock et al., 2019; Chera et al., 2009) and this mechanism might be involved in Xenopus tail regeneration. Another unexplored possibility would be the recycling of components from dying cells to re-structure the regenerating tail.”

Also, because Lysosensor can detect low pH lysosomes in macrophages it is unclear if they are measuring apoptotic cells or macrophage recruitment. This should be discussed.

As we detect higher apoptosis levels following Clodrosome injection or Spib gene knock-out regeneration-competent tadpoles, it is unlikely that we are detecting recruited macrophages as these cells are depleted in these conditions. Nonetheless, we agree that Lysosensor may not be the most specific method to detect apoptotic cells. We discuss limitations of our approach in detail in the discussion section:

Page 9 line 257-264

© 2020. Published by The Company of Biologists under the terms of the Creative Commons Attribution License (https://creativecommons.org/licenses/by/4.0/).

"In this work, we detected apoptosis using Lysosensor which labels cells based on cytoplasmic acidity, a hallmark of apoptotic cells. We tested the specificity of this approach by comparing it to published Caspase-3 immunohistochemistry analysis (Tseng et al., 2007), and tested it against an apoptosis inhibitor. As Lysosensor is sensitive to pH, we may have recorded high pH containing cells other than apoptotic cells, including macrophages. However, as we detect more Lysosensor signal upon different myeloid lineage depletion protocols and we do not detect this signal as sparse distinct cells, it is unlikely that it originates from recruited macrophages."

Fig S2 would benefit from Inset boxes. Numbering in high magnification images are not clear.

We have enlarged the images so that the reader can better distinguish the shape and labelling of SLURP1L:GFP cells.

Second decision letter

MS ID#: DEVELOP/2019/185496

MS TITLE: The myeloid lineage is required for the emergence of a regeneration permissive environment following Xenopus tail amputation

AUTHORS: Jerome Jullien, Can Aztekin, Tom Hiscock, Richard Butler, Francisco De Jesus Andino, Jacques Robert, and John Gurdon
ARTICLE TYPE: Research Article

I am happy to tell you that your manuscript has been accepted for publication in Development, pending our standard ethics checks.

Reviewer 1

Advance summary and potential significance to field

Please refer to previous assessment

Comments for the author

Dear Authors, thank you for addressing the few points I had made at the previous review.

Reviewer 2

Advance summary and potential significance to field

The authors have made thoughtful and thorough responses to the concerns raised by each of the three reviewers. The manuscript looks to be more thorough and brings up possible alternative conclusions and considerations based upon the results. The contribution to the field is field is strong and will yield considerable interest in the field.

Comments for the author

I do not have further comments on the manuscript. The authors responded thoroughly to all concerns raised by the reviewers.

© 2020. Published by The Company of Biologists under the terms of the Creative Commons Attribution License (https://creativecommons.org/licenses/by/4.0/).

Reviewer 3

Advance summary and potential significance to field

The authors tie together several known myeloid related functions in a regeneration model that will be helpful for some in the community.

Comments for the author

The authors have done a good job of tempering some of their conclusions and making the data more interpretable. They have also included comments into the discussion on many of the biological discrepancies in the data that put these in the correct context. Given these revisions, I feel this seems appropriate for publication in development.

© 2020. Published by The Company of Biologists under the terms of the Creative Commons Attribution License (https://creativecommons.org/licenses/by/4.0/).
